# Spatial Profiling of Nuclear Receptor Transcription Patterns over the Course of *Drosophila* Development

**DOI:** 10.1534/g3.113.006023

**Published:** 2013-07-01

**Authors:** Ronit Wilk, Jack Hu, Henry M. Krause

**Affiliations:** Department of Molecular Genetics, The Terrence Donnelly Centre for Cellular and Biomolecular Research, University of Toronto, Toronto, ON, Canada, M5S 3E1

**Keywords:** *Drosophila*, development, expression, nuclear receptor, subcellular

## Abstract

Previous work has shown that many of the 18 family members of *Drosophila* nuclear receptor transcription factors function in a temporal hierarchy to coordinate developmental progression and growth with the rate limiting process of metabolism. To gain further insight into these interactions and processes, we have undertaken a whole-family analysis of nuclear receptor mRNA spatial expression patterns over the entire process of embryogenesis, as well as the 3rd instar wandering larva stage, by using high-resolution fluorescence *in situ* hybridization. Overall, the patterns of expression are remarkably consistent with previously mapped spatial activity profiles documented during the same time points, with similar hot spots and temporal profiles in endocrine and metabolically important tissues. Among the more remarkable of the findings is that the majority of mRNA expression patterns observed show striking subcellular distributions, indicating potentially critical roles in the control of protein synthesis and subsequent subcellular distributions. These patterns will serve as a useful reference for future studies on the tissue-specific roles and interactions of nuclear receptor proteins, partners, cofactors and ligands.

Nuclear receptors (NRs) comprise a large family of highly conserved transcription factors with a unique ability to monitor lipophilic hormone distributions and to convert this information into coordinated transcriptional responses. Humans have 48 NRs and *Drosophila* only 18 (reviewed by Thummel 2001; King-Jones and Thummel 2005). Importantly, flies have representatives of all NR subclasses without the duplication seen in humans. Where tested, orthologues appear to have retained the same general properties and functions, including structures, ligands, and target genes (Pardee *et al.* 2011). Hence, new advances in our understanding of insect NR functions will continue to provide highly relevant and important insights into human NR regulation and functions.

With their ability to control metabolism, growth, development, reproduction, and associated behaviors, NRs feature in virtually all important physiological functions. Consequently, misexpression or inappropriate activity results in numerous devastating diseases, including metabolic, autoimmune and gender based disorders, as well as many neurological disorders and most cancers (Gollamudi *et al.* 2008; Vacca *et al.* 2011; Anbalagan *et al.* 2012). A system-wide understanding of the expression patterns of these genes would provide many new insights into their roles in normal and disease processes. Despite their importance, however, relatively few system-wide expression studies have been performed on the NR gene family. In vertebrates, the most informative has been a polymerase chain reaction (PCR)-based analysis of adult tissues. However, little attention has been given to embryonic or spatial expression patterns.

Although *Drosophila* has the advantage of having far fewer NRs and tissues to contend with, even less is known about the full 18-member family. As with vertebrates, expression analysis has been limited to northern blotting or PCR-based analyses, and only on whole animals, thereby providing only a quantitative average of all spatial expression patterns. A system-wide analysis of NR activity patterns has also been conducted in flies, but although this suggested likely sites of ligand and cofactor presence, it does not necessarily imply sites of NR gene expression (Palanker *et al.* 2006). In addition, the analysis was only successful for the NRs that function as transcriptional activators (approximately half).

In this analysis, we performed a relatively high resolution and sensitive analysis of *Drosophila* NR spatial expression patterns using tyramide signal-amplified fluorescence *in situ* hybridization (TSA-FISH). We follow the entire course of embryogenesis as well as the penultimate stage of larval development. A large degree of overlap is seen between expression patterns at these different stages of development, with the most common sites of expression being those involved in metabolic, endocrine and behavioral control. Temporal and spatial overlaps in these expression patterns will help define their combinatorial and hierarchal relationships and roles. Remarkably, despite their role as transcription factors, and the abilities of the encoded proteins to relocate from the site of production to the nucleus, the majority of NR transcripts were seen to exhibit numerous and complex patterns of subcellular distribution, suggesting new and important roles in the regulation of these genes.

## Materials and Methods

### Preparation and *in situ* hybridization of *Drosophila* embryos

Oregon-R (wild type) embryos were collected, fixed, and hybridized as described in (Wilk *et al.* 2010). All the probes used in this study were produced as described in the same paper. Oligos used for PCR amplification are shown for each transcript in Supporting Information, Table S1. In the case of *NOS*, a two-step nested PCR was performed (see Table S1 for details).

### Dissection, fixation, and *in situ* hybridization of wandering 3rd instar larval tissues

Preparation, fixation, and *in situ* hybridization were performed as described (Wilk *et al.* 2010) with one modification: quenching of endogenous horseradish peroxidase was performed for 30 min in 0.3% H_2_O_2_ in PBS. Fresh solution was substituted at 15-min mark.

### Double green fluorescence protein (GFP) antibody and *in situ* hybridization in 3rd instar larval tissues

To detect GFP expression on the endoplasmic reticulum (ER), we used a

*w;Sec61-alpha-GFP* line kindly provided by Dr. Julie Brill. Again, we followed the published FISH protocol (Wilk *et al.* 2010) with the following modifications and additions:

- The first fixation was performed for 10 minutes in 2% paraformaldehyde in phosphate-buffered saline (PBS) with 0.3% Triton X and no picric acid.
- Endogenous horseradish peroxidase quenching was performed in 0.3% H_2_O_2_ for 15 min, with one change of solution at the 7-min mark.
- Permeabilization of the tissues was done using 80% acetone for 5 min at −20ºC.
- The second fixation was in 2% paraformaldehyde for 10 min.
- After the second fixation, samples were blocked with PBS-Tween 20 (PBT) buffer containing 0.33 units/mL of RiboLockerTM RNase inhibitor (cat. no. E00381l; Fermentas) for 30 min.
- Anti GFP antibody (rabbit polyclonal anti-GFP antibody Abcam 290) was applied with 1/350 dilution in PBT buffer with RiboLocker, incubated at room temperature (RT) for 1 hr. Four washes were done at RT with PBS for 5 min.
- Hybridization time was reduced to 8 hr at 52º.
- Secondary antibody (code number 711-095-152, FITC-conjugated Donkey anti-Rabbit IgG; Jackson ImmunoResearch Laboratories) used to recognize the GFP primary antibody was added after *tyramide signal amplification* staining and subsequent washes. Dilution was 1:125 in PBT buffer, followed by four washes at RT in PBS for 5 min each on a Nutator.

### Cluster trees

All data obtained from images was summarized as a tab-delimited text file as required by the Gene Cluster 3.0 software. After clustering, the data were acquired using Java TreeView software, and figures were created with a combination of Java TreeView images and Adobe Illustrator.

### Microscopy and imaging

All images were acquired with a Leica DMRA2 microscope (dry 20× and 40× lenses for most images, 63× oil for Figure 10) and a Q-Imaging Retiga EX camera, using Openlab 3.1.7 software running on an Apple iMac computer. All images are pseudo-colored using Adobe Photoshop with 4’,6-diamidino-2-phenylindole shown in red and RNA in light blue (except for Figure 10). All figures were created with a combination of Adobe Illustrator and Adobe Photoshop. All images are archived in internal lab-databases (one for embryos one for larvae) and are publicly available on FlyFISH (http://fly-fish.ccbr.utoronto.ca).

## Results

### Expression and localization of NR transcripts during embryogenesis

**Figure 1:**
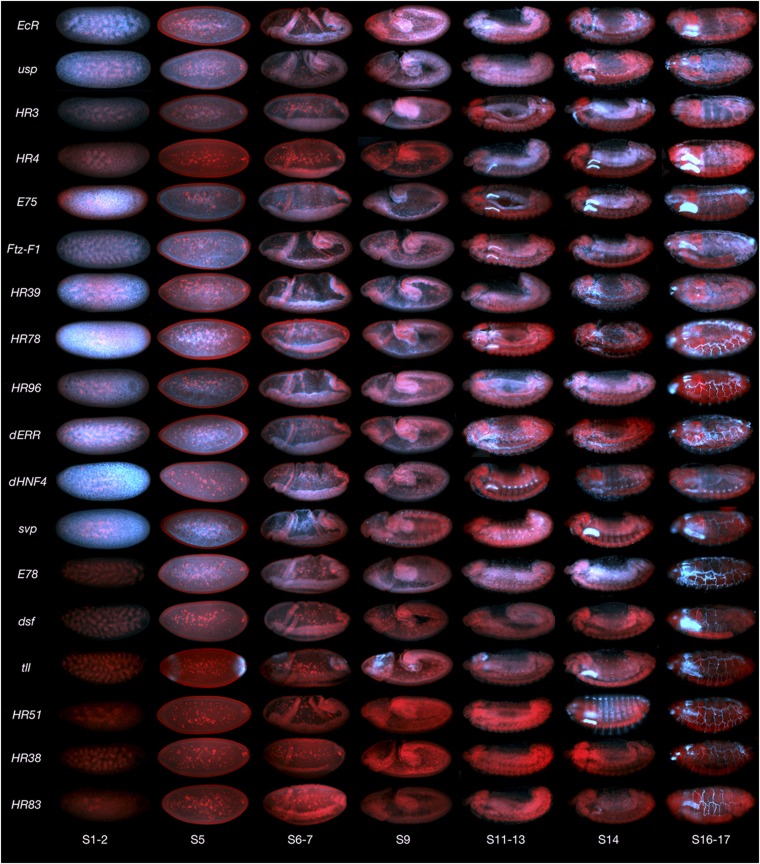
Composite of all NR expression patterns over the course of embryogenesis. Embryos are oriented anterior to the left, and dorsal up. Nuclei are red, and RNA is blue.

**Figure 2:**
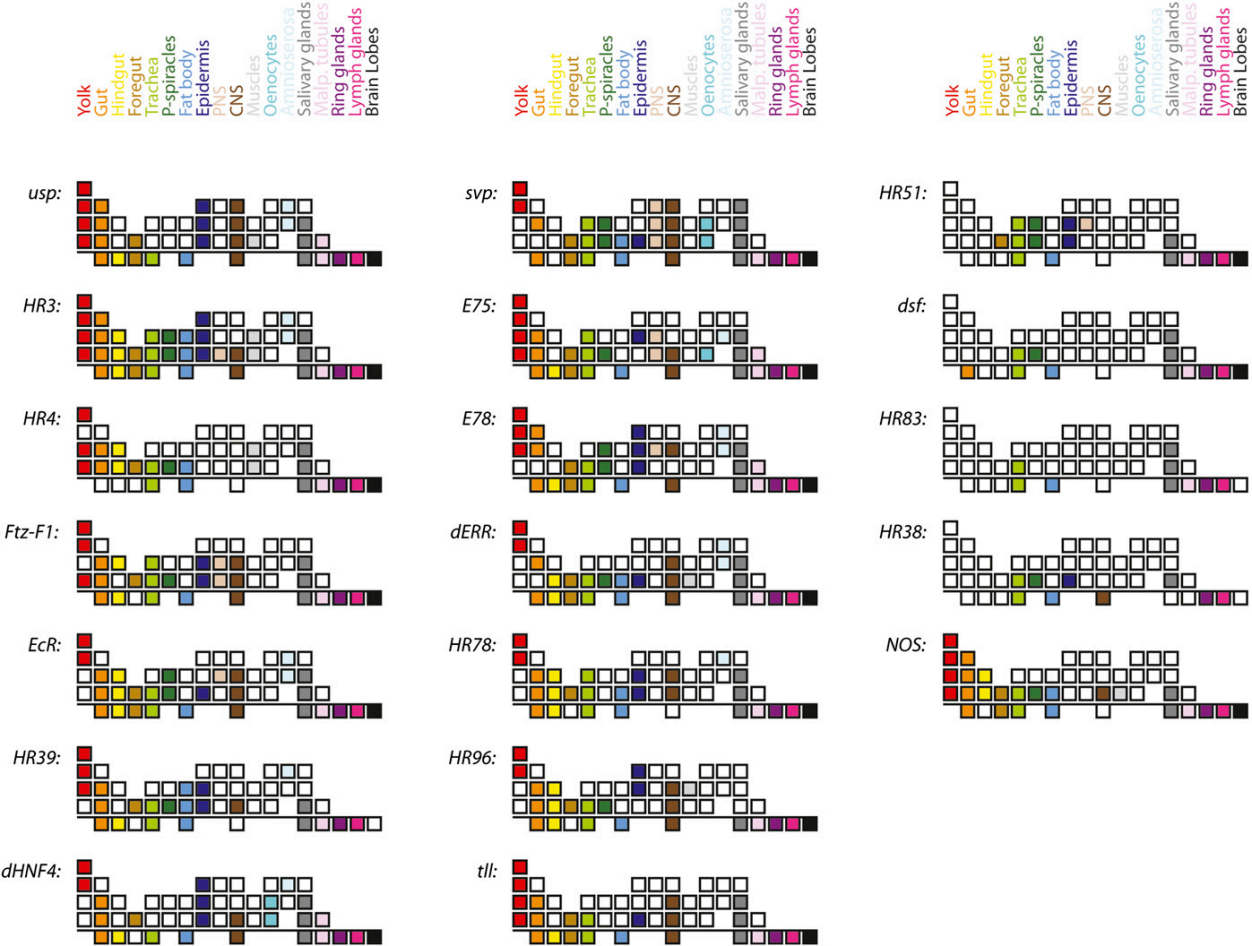
Graphic summary of NR tissue distributions. Stages of development and individual tissues are indicated as a series of boxes. For each NR, the top four rows indicate various tissues during different stages of embryonic development (Stages 1−5, 6−10, 11−14, and 15−17, respectively) and the bottom row indicates tissues of wandering 3rd instar larva. Each tissue is represented by a unique color as specified in the color key at the top. NOS transcript distributions are also shown for comparison (bottom left).

Whole-mount *in situ* hybridization to monitor the expression patterns of the 18-gene NR family (listed in Table S1) in embryos was performed using TSA-FISH, as described previously (Lecuyer *et al.* 2007, 2008). To maximize coverage and efficiency, DIG-labeled RNA probes that recognize all or most NR splice variants were designed and produced (Table S1). A comparative overview of the expression patterns derived from embryos is shown in Figure 1 (blue: RNA, red: DNA). Greater-resolution and additional images can be found on the Fly-FISH in situ hybridization database (fly-fish.ccbr.utoronto.ca), and the figures herein. A color-coded chart summarizing stages and tissues of expression is shown in Figure 2, with embryonic stages graphically represented by the first four rows of each composite (subgrouped into stages 1−5, 6−10, 11−14, and 15−17 respectively).

**Figure 3:**
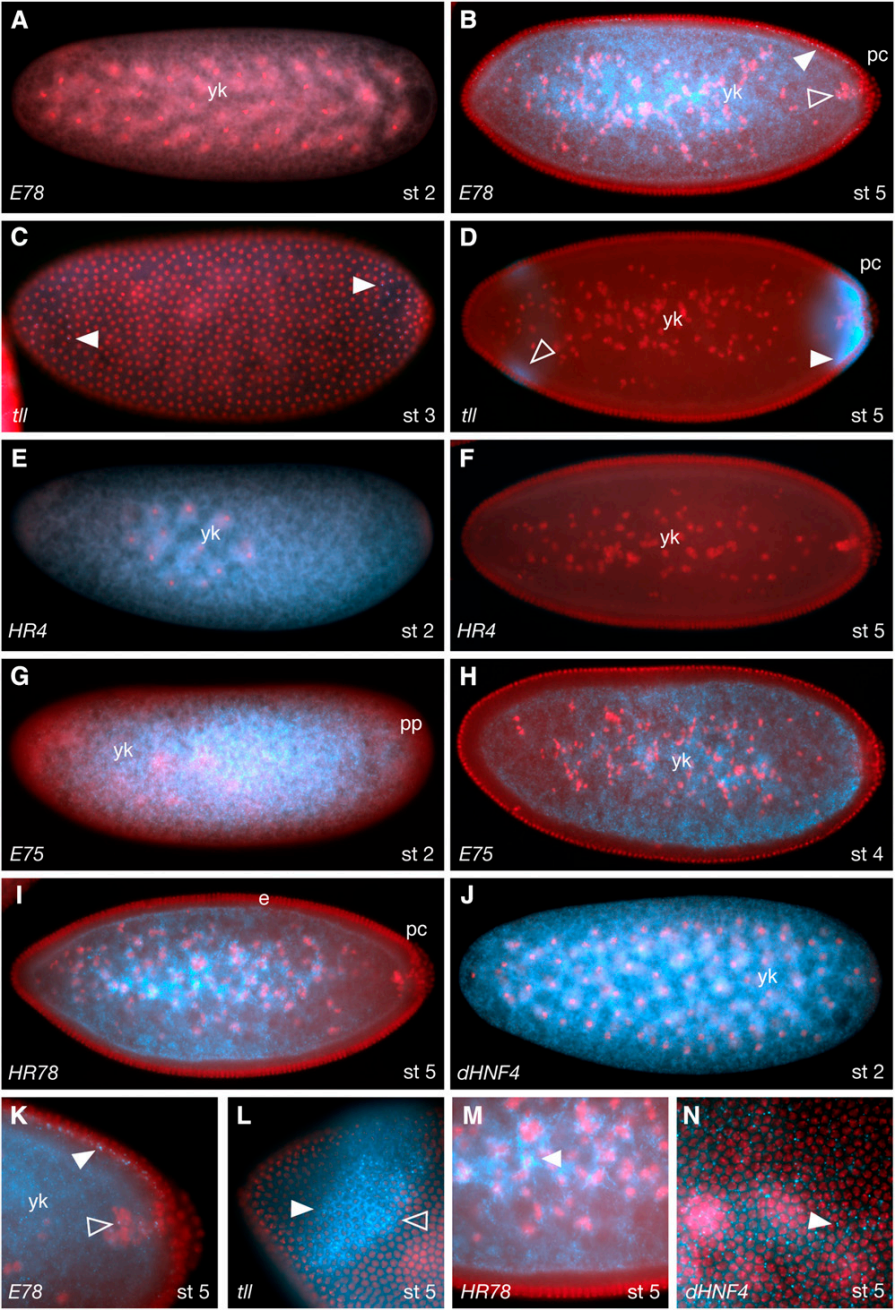
NR expression patterns during early embryogenesis. (A, B) *E78* in stage 2 and 5 embryos respectively, showing no maternal RNA (A), and zygotic transcription during stage 5 (B, K). White arrowheads indicate nascent transcripts in epithelial nuclei, and clear arrowheads in yolk nuclei. (C, D) *tll* in stage 3 (C) and 5 (D, L) embryos. Arrowheads show basal enrichment. Focus on the plane of the nuclei within the anterior *tll* stripe shows both nascent (white arrowhead in L) and cytoplasmic (clear arrowhead in L) transcripts. (E, F) *HR4* in stage 2 and 5 embryos, respectively. Maternally provided transcripts are completely degraded by stage 5 with no zygotic replenishment. (G, H) *E75* in stage 2 and 4 embryos, respectively. Transcripts are rapidly removed from the posterior plasm (pp) and the peripheral cortex. (I, M) *HR78* in a stage 5 embryo. The arrowhead in (M) indicates enrichment around the yolk nuclei. (J, N) *dHNF4* in stage 2 and 5 embryos showing maternal (J) and zygotic (N) expression. Panels K−N are greater-magnification images of embryos stained for the receptors indicated.

As seen in Figure 1, the majority of NRs are expressed maternally. Exceptions include *E78*, *dsf*, *tll*, *HR51*, *HR38*, and *HR83*. Notably, although not expressed maternally, *E78* soon achieves a very similar pattern via zygotic transcription that begins at stage 4. Also notable is that although maternal transcripts are distributed relatively uniformly early, both the maternal and zygotically expressed transcripts are quickly excluded from the syncitial epithelial layer. In the case of *E78*, this is despite being transcribed, in part, within these peripheral nuclei (Figure 3, A, B, and 3K). All maternally expressed transcripts are also excluded from the pole cells as they bud off from the posterior. In most cases, these stage 3−6 transcripts become enriched in the central yolk area near or around the yolk nuclei (Figure 3). The exception at this stage is *tll*, which is zygotically expressed and localized in anterior and posterior stripes, with some basal enrichment below the syncitial nuclei layer (Figure 3, C, D, and L).

**Figure 4:**
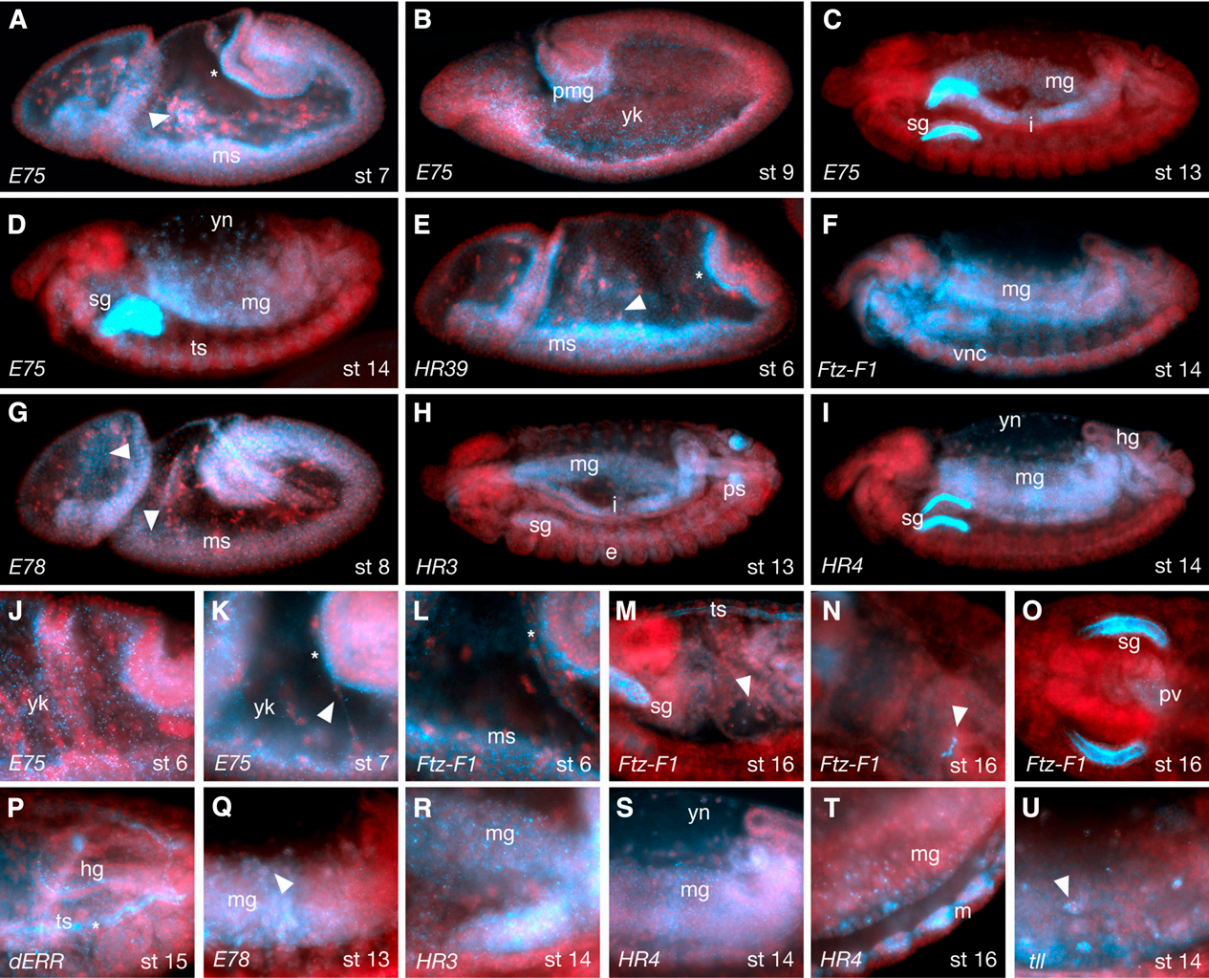
NR expression in the mesoderm and gut. (A−D, J, K) *E75* in stage 6−14 embryos. During stages 6 and 7, *E75* mRNA is enriched in foci within the yolk (yk), enriched basally in the epithelial layer (asterisks in A and K), perinuclearly around yolk nuclei (arrowhead in A). Arrowhead in K) indicates localization on filopodia-like processes. At germband extension (B), *E75* is seen in posterior midgut cells (pmg) and continues to be enriched in yolk foci (yk). After germband retraction (C, D) expression is seen in the developing midgut (mg), interstitial cell precursors (i), tracheal system (ts), salivary glands (sg), and yolk nuclei (yn). (E) During gastrulation, *HR39* is enriched in mesoderm (ms), basal epithelia (asterisk) and around yolk nuclei (arrowhead). (F, L−O) *Ftz-F1* transcripts are enriched in the mesoderm (ms) and basal epithelia during gastrulation (asterisk in L). Later (F), it is expressed in ventral nerve cord (vnc), midgut (mg), salivary glands (sg in M), yolk nulcei (arrowhead in M), tracheal system (ts in M), and the border between hindgut and midgut (arrowhead in panel N), as well as the proventriculus (pv in panel O). (G, Q) *E78* in stage 8 and 13 embryos respectively, showing enrichment in mesoderm (ms) in small cytoplasmic foci (arrowheads in G), and around yolk nuclei (arrowhead in Q). (H, R) *HR3* expression in midgut (mg), salivary glands (sg) and posterior spiracles (ps). (I, S, T) *HR4* probe in stage 14 and 16 embryos showing expression in midgut (mg), hindgut (hg), yolk nuclei (yn), salivary gland lumen (sg) and somatic muscles (m in panel T). (P) *dERR* probe at stage 15, showing widespread expression with enrichment in the hindgut (hg) and tracheal system (ts) lumen (asterisk). (U) *tll* transcripts inside yolk nuclei (arrowhead) of a stage 14 embryo. Panels J−U are greater-magnification images of embryos stained for the receptors indicated.

As gastrulation proceeds, the interiorly localized mRNAs described previously continue to be localized to yolk plasm as well as the forming mesoderm (Figure 4). In the mesoderm, transcripts are highly enriched towards the inner yolk plasm (Figure 4, A, B, and E). Strong zygotic expression is now observed in and around the yolk nuclei for all previously expressed genes (Figure 4, A and E). In some cases, transcripts in the yolk can also be seen bound to filopodia-like processes (Figure 4L, arrowhead). By stages 10−12, these patterns largely give way to expression in the forming midgut (Figure 4), which encloses around the yolk and yolk nuclei. As other parts of the gut (*e.g.*, hindgut, proventriculus, esophagus) begin to form, a number of NR transcripts also come to be expressed there; the only NRs not expressed in the gut are *dsf*, *HR83*, and *HR38* (also see Figure 2 and Table S2). The NRs E78, HR3, and dHNF4 have previously been reported to be active *in vivo* in the midgut at these same stages (Palanker *et al.* 2006). In addition to midgut, *HR3*, *HR4*, *Ftz-F1*, *EcR*, *dERR*, *HR78*, and *HR96* are also expressed in the embryonic hindgut (*e.g.*, Figure 4P). Interestingly, many NRs were observed in the imaginal cells that form the junction of the midgut and hindut (*e.g.*, Figure 4N and Figure S1). Given that Fushi tarzu, which is the heterodimer partner of 〈Ftz-F1 (Copeland *et al.* 1996; Guichet *et al.* 1997; Schwartz *et al.* 2001), is also specifically expressed in these cells (Krause *et al.* 1988), it may be this particular *Ftz-F1* splice form that is detected by our probe. *Ftz-F1*, *tll*, *E78*, and *usp* were also observed in anterior gut regions such as the proventriculus (*e.g.*, Figure 4O and Figure S1).

**Figure 5:**
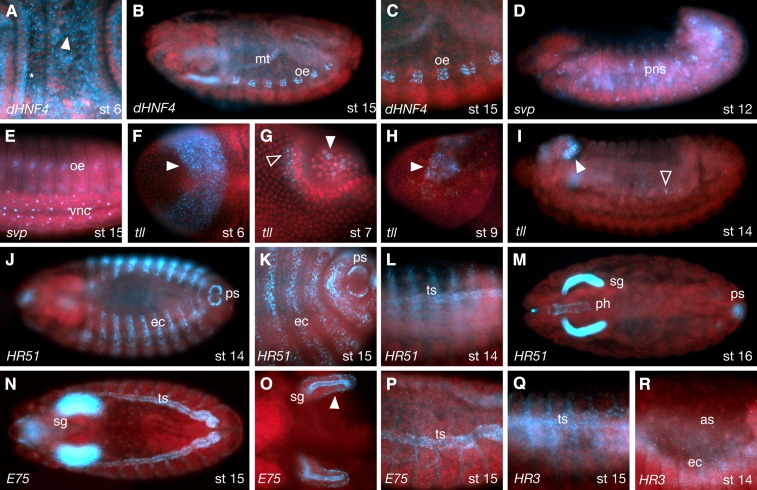
Other sites of NR expression. (A−C) *dHNF4* expression and localization seen in cytoplasmic foci and enriched basally at stage 6 (arrowhead and asterisk in panel A respectively), oenocytes (oe) and Malpighian tubules (mt) during stage15 (B−C). (D−E) *svp* in stage 12 and 15 showing expression the peripheral nervous system (pns) and oenocytes (oe) and localization in ventral nerve cord (vnc) nuclei. (F−I) *tll* expression I and localization during stages 6−14. (F) Strong anterior stripe at stage 6 with nascent and cytoplasmic puncta (arrowhead). (G) shows puncta in posterior (clear arrowhead) and pole cell expression with perinuclear localization (white arrowhead). (H, I) Head expression resolves to a subset of brain primordia (white arrowheads). PNS (Peripheral Nervous System) expression (clear arrowheads) is also seen. (J−M) *HR51* in stage 14−16 embryos showing expression in ectodermal stripes (ec) and posterior outside part of the spiracles (ps). (L) Tracheal progenitor cell staining, limited predominantly to the dorsal trunk, and later (M) in the pharynx (ph) and the salivary glands (sg). (N−P) *E75* at stage 15 is clearly expressed in the developing tracheal system (ts), along with strong luminal surface localization and basal puncta (white arrowhead in O) in salivary gland cells. (Q, R) Tracheal staining is also seen with the *HR3* probe as well as amnioserosa (as) nuclei and ectodermal (ec) foci. Panels A, C−H, K, L, and O−R are higher-magnification photos of embryos stained for the receptors indicated.

Other common sites of NR expression, starting at around mid-embryogenesis, are the trachea, salivary glands, fat body, amnioserosa, and nervous system (Figures 2, 4, and 5). The dynamic nature of some of these expression patterns is consistent with earlier observations of hierarchal interactions. Note, for example, the expression patterns of *HR3*, *HR4*, *E75*, *Ftz-F1*, and *EcR* in the salivary glands (Figure 1). Sites of expression that are less common include muscle (*dERR*, *HR4*, *HR3*, *HR96*, and *usp*, *e.g.*, Figure 4T), oenocytes (*dHNF4*, *svp*, and *E75*; Figure 5, B, C, and E) and the peripheral nervous system (*svp*, *E75*, *E78*, *HR3*, *Ftz-F1*, *EcR*, and *HR51*; Figure 5D). Interestingly, *svp* transcripts are often retained within the nuclei of neurons in the central nervous system (CNS), peripheral nervous system (PNS), and oenocytes (Figure 5E).

The unique early expression of *tll* also gives rise to patterns of CNS and brain expression in the anterior and a subset of germ line cells in the posterior (Figure 5, F−I). *HR51* transcripts are also unique, with initial expression only after germ band retraction in segmental stripes (Figure 5, J−M). Later, strong salivary gland expression occurs, along with expression in the pharynx and posterior spiracles. *dHNF4*, and to a lesser extent *usp*, *E75*, and *E78*, are the only NR transcripts notably enriched in Malpighian tubules during embryogenesis (*e.g.*, Figure 5B). Notably, most NR transcripts continue to exhibit some form of nonrandom subcellular distribution in these later tissues and stages (see Table S3).

### NR expression during late 3rd instar larval development

To achieve further insight into tissue-specific and developmental NR functions and to obtain greater-resolution subcellular images, we decided to include the larger 3rd instar larvae in our spatial analyses of expression patterns. NRs play a major role in choreographing the switch from larval growth to metamorphosis at the end of the 3rd instar, much as they do during the initiation of puberty in adults in humans. We focused on the last hours of the 3rd instar by using wandering 3rd instar larvae that were no longer feeding. The same probes were used, together with a recently described *FISH* protocol for larval tissues (Wilk *et al.* 2010) with some minor modifications (see *Materials and Methods*).

**Figure 6:**
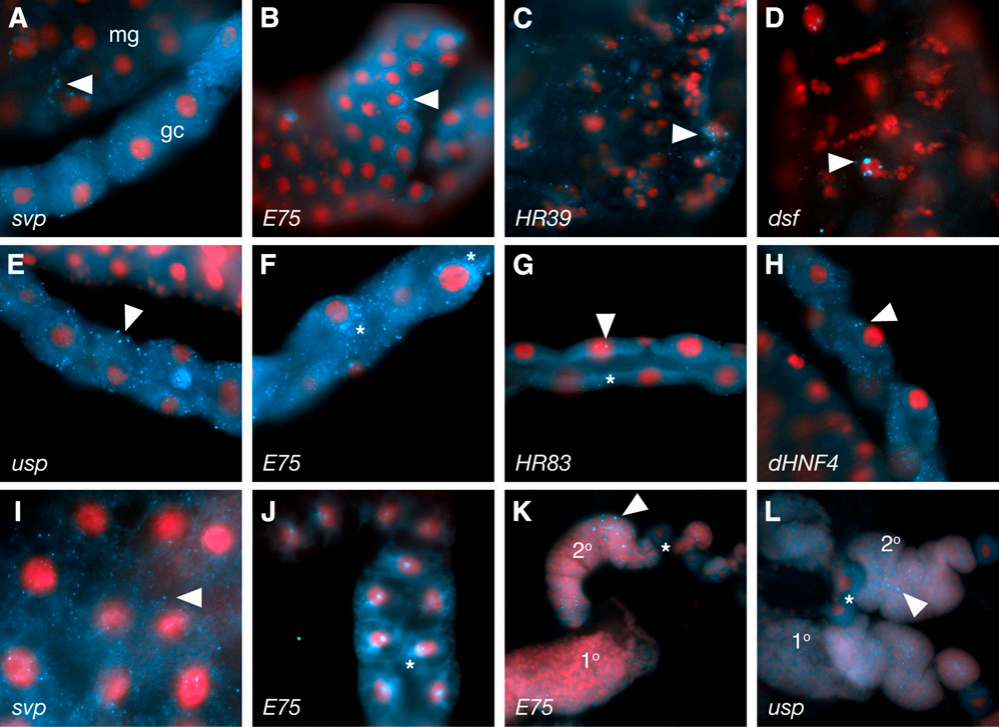
3rd instar larval expression in the gut, Malpighian tubules and fat body. Intestine (A-D), Malpighian tubules (E−H) fat body (I, J) and lymph glands (K, L). NRs probed are indicated at the lower left of each panel. Arrows in (A) and (C) indicate cytoplasmic foci. “Half–moon” perinuclear expression of *E75* is shown in (B) (arrowhead), and sub-nuclear foci for *dsf* in D) (arrowhead). In the Malpighian tubules, large cytoplasmic foci are common (arrowheads in E, G, H). *E75* again forms a “half–moon” perinuclear pattern (asterisks in F). *HR83* is seen in relatively few foci (asterisk in G). (I) Typical fat body localization as revealed by *svp*, as opposed to *E75* (J), which once again exhibits perinuclear localization (asterisk). (K, L) Lymph gland primary (1º) and secondary lobes (2º) show ‘foci’ between nuclei (arrowheads in K , L), as well as uniformly diffuse staining at varying levels (L). Pericardial cells are marked by an asterisk.

**Figure 7:**
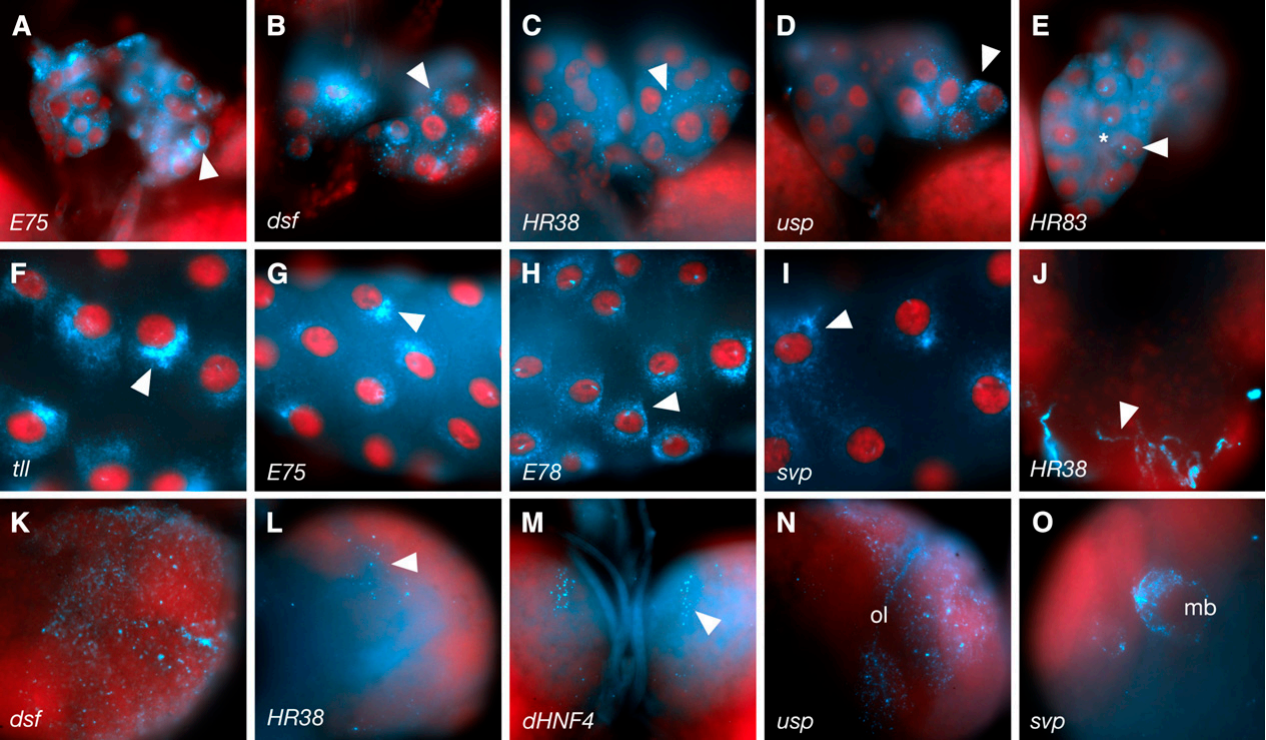
3rd instar expression in the prothoracic gland, salivary gland, lymph gland and brain. Typical expression patterns are shown for the prothoracic gland (A−E), salivary glands (F−I), ventral nerve cord (J), and brain (K-O). NR identities are indicated at the lower left of each panel. Subcellular distributions in the prothoracic gland vary from perinuclear (A) to cytoplasmic foci in various sizes and distributions (B−D, arrowheads) or diffuse cytoplasmic (asterisk in E). In the salivary glands, *tll* (F) and *E75* (G) are localized in tight “half-moon” perinuclear patterns, whereas *E78* (H) exhibits more diffuse perinuclear localization (arrowhead in H). Conversely, *svp* is distributed relatively uniformly, with some enrichment around nuclei (I; arrowhead). (J) *HR38* is subcellularly localized in ventral nerve cord axons (arrowhead). Brain lobe patterns (K-O) are highly variable, including expression in optic lobes (N; ol) and mushroom bodies (O; mb).

As with embryos, we found that most NRs are expressed in tissues that are essential for metabolite intake, storage, metabolism and excretion, including parts of the gut, Malpighian tubules and fat body. NR expression was also highly enriched in endocrine and secretory organs such as the prothoracic gland, portions of the brain, fat body, oenocytes, salivary glands and lymph glands. These are described further below and in Figures 2, 6, 7, Table S2, and Table S3.

As expected, and as seen during embryonic development, the transcripts of 15 NRs are also present in the larval midgut and/or hindgut, as well as the more anterior proventriculus and gastric caeca (Figure 6, A−D; Table S2). In most cases, the transcripts tend to accumulate in small distinct cytoplasmic foci (Figure 6, A and C). Interestingly, some *NR* transcripts, such as those of *E75* (Figure 6B) form a full or partial halo around the nuclei. All NR transcripts, with the exception of *HR38*, are also expressed in the Malpighian tubules, which serve as the larval kidneys. Here, transcripts also tend to be subcellularly localized within cytoplasmic foci that vary in size and number (Figure 6, E−H). Interestingly, E75 is again found as a partial halo around the nuclei (asterisk in Figure 6F).

The *Drosophila* fat body is the primary storage site for a variety of dietary and metabolic lipids. It is also the primary secretory organ of the larva with many functions in common with the human liver. Interestingly, we found that all 18 *Drosophila* NRs are expressed in the fat body during wandering 3rd instar larval stages (Figure 6, I and J; Figure 2; Table S2). Once again, most of the expressed transcripts localize subcellularly to discrete cytoplasmic foci (*e.g.*, Figure 6I), and as seen in the gut and the Malpighian tubules, some transcripts such as those of *E75* were once again localized in perinuclear “half-moon” patterns (asterisk in Figure 6J).

Another tissue in which all 18 NRs were found to be expressed is the lymph gland (*e.g.*, Figure 6, K and L). As in vertebrates, the lymph gland is where hematopoiesis occurs, and hemocytes produced there are essential for pathogen encapsulation (Roehrborn 1961; Jung *et al.* 2005; Krzemien *et al.* 2010). During larval stages, the lymph gland consists of a pair of primary lobes (1º) and smaller secondary lobes (2º) intercalated by pericardial cells (asterisks in Figure 6, K and L). Most NRs are found throughout the glands with clear foci between the cell nuclei (arrowheads in Figure 6, K and L) some in higher levels (*e.g.*, *usp*, Figure 6L). *HR3* and *HR78* were exceptions, with no foci detected (Figure S2).

The ring gland, which is found above and anterior to the larval brain lobes and behind the lymph gland, was also found to express all 18 NRs at these stages of development (Figures 2 and 7, A−E). Notably, the majority of this expression occurs in the prothoracic gland, which makes up the largest portion of the ring gland, and which is the main site of larval ecdysone production. In most cases (15 of 18) the RNA is again localized to small cytoplasmic foci (Figure 7, B−D, Table S3). Once again, *E75* tends to form “half-moon” halos around the nuclei (Figure 7A). Others, such as *usp*, form a more diffuse perinuclear halo (Figure 7D).

The larval salivary glands undergo extensive morphological and compositional changes prior to morphogenesis, as they get ready to switch from aiding digestion to secreting the glue proteins that are used to attach the pre-pupae to appropriate surfaces. These changes are also controlled by ecdysone. We found that the transcripts of all NRs, with the exception of *HR38*, are expressed in the salivary glands at this stage (Figure 2). Here, “half–moon” perinuclear distributions were clearly observed for a number of transcripts (*e.g.*, Figure 7, F−I; see Table S3 for more details).

Below the prothoracic and lymph glands, most NRs (*HR39* and *HR83* excepted) also are present in portions of the brain and/or ventral nervous system (Figure 7, J−O). However, unlike the tissues above, each of these patterns was unique, encompassing different subsets of neurons. Of these, *dsf* exhibits the broadest distribution (Figure 7K) and *HR38* and *dHNF4* the most restricted patterns (Figure 7, L and M). *E78*, *tll*, *usp*, *dsf*, *svp*, *E75*, *HR78*, and *Ftz-F1* are all expressed within portions of the optic lobes (*e.g.*, Figure 7N), and *HR51* and *svp* appear to be expressed in the mushroom bodies (*e.g.*, Figure 7O). Notably, in the ventral nerve cord, *HR38* transcripts are highly abundant within neuronal axons (Figure 7J).

### Expression of NRs in imaginal discs

**Figure 8:**
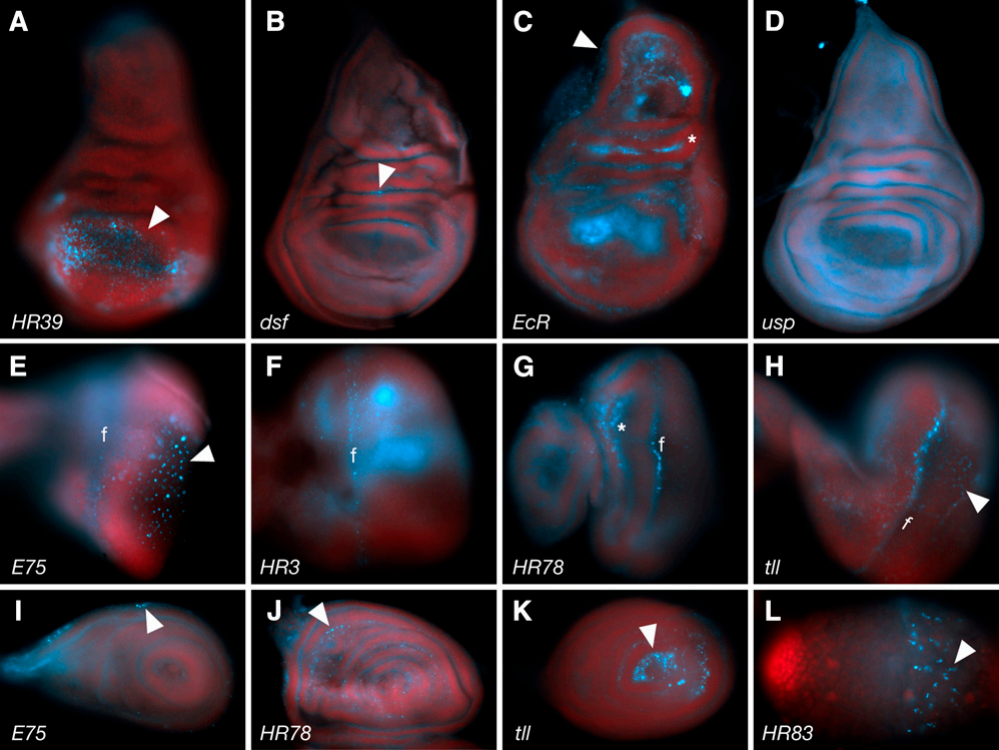
3rd instar larval expression in imaginal discs. (A-D) Examples of expression patterns in wing discs. *HR39* is enriched dorsally in the wing pouch (A; arrowhead). *dsf* expression is limited (B; arrowhead ), whereas *EcR* is expressed throughout most of the disc (C) with enrichment in the hinge (asterisk) and body wall (arrowhead). *usp* expression is also widespread, but unlike the others is diffuse (D). In the eye disc (E-H, the morphogenetic furrow (f) and differentiating photoreceptors (arrowheads) are common site of expression. (I-K) Examples of leg disc expression patterns. (L) Larval testis with *HR83* expressed in somatic cyst cells (arrowhead).

The majority of the aforementioned larval tissues described are polyploid and do not survive into adulthood. This is not the case for imaginal discs, which transform during metamorphosis into much of the adult fly structures. Although our larval preparation and *FISH* protocols did not manage to collect these tissues in a consistent and comprehensive fashion, we did document a number of eye, leg and wing imaginal disc expression patterns (See Table 1 and Figure S1; last three columns). Some of the more notable patterns are shown in Figure 8.

**Table 1:** Summary of imaginal disc expression

Gene	Eye Disc	Wing Disc	Leg Disc
*EcR*	++	+	++
*usp*	++	++	++
*HR3*	++	+/−	++
*HR4*	++	++	++
*E75*	++	++	+
*FtzF1*	+/−	−	−
*HR39*	+	+	++
*HR78*	+	−	+
*HR96*	−	+	−
*dERR*	−	−	−
*dHNF4*	++	++	++
*svp*	++	+	++
*E78*	++	++	++
*dsf*	+	+/−	+/−
*tll*	++	+	+
*HR51*	−	++	+
*HR38*	+	−	−
*HR83*	−	−	+
*Nos*	++	++	++

++, high levels; +, low; +/−, low/inconsistent; −, no expression.

### Colocalization of NR transcripts with ER

**Figure 9:**
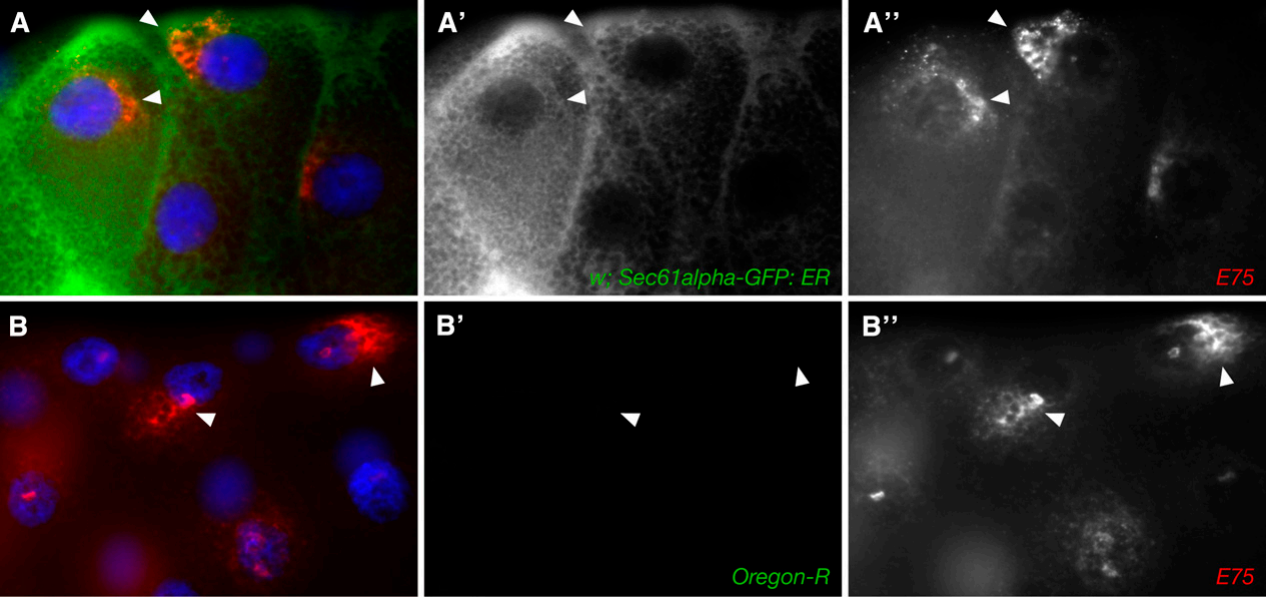
Perinuclear *E75* expression overlaps with rough ER. (A) Third instar salivary gland cells are shown stained for *E75* transcripts (red) and a marker of rough ER (Sec61alpha-GFP; green). Nuclei are blue. Individual channels for *E75* (A′) and Sec61alpha-GFP (A′′) are shown to the right. Arrowheads indicate locations where overlap is clearest. (B) Same experiment done in an Oregon R (wild type) larval salivary gland as a negative control.

The distribution of several NR transcripts in close proximity to the nuclear membrane, as well as the particulate nature of their distributions, strongly suggested some form of association with ER. We explored this association by performing double labeling with NR transcript FISH and Immunofluorescent detection of GFP-tagged ER proteins. As seen in Figure 9, *E75* transcripts show a clear overlap with perinuclear regions of *Sec61 alpha*-GFP, a protein that specifically marks ER. No significant overlap was observed for *E75* transcripts and other organelle markers (data not shown).

### Expression of nitric oxide synthase (NOS)

Nitric oxide (NO) gas, produced by the enzyme NOS, has been shown to play important roles in many NR-regulated processes, including cell and tissue growth, metabolism, inflammation, circadian rhythm, and metamorphosis. In flies, NO has been shown to interact with E75 and HR51 *in vitro* (Reinking *et al.* 2005, Aicart-Ramos *et al.* 2012). NO has also been shown to coordinate ecdysone production via regulation of E75 activity in the prothoracic gland, with subsequent effects on downstream NRs in the ecdysone pathway hierarchy (Caceres *et al.* 2011). Although *NOS* is known to be expressed in the prothoracic gland and imaginal discs in larvae, its expression and requirement in other tissues is not well documented. Consequently, a careful analysis of *NOS* spatial expression patterns was deemed relevant and included in this study. The probe used recognizes the majority of (8 of 10) alternative NOS transcripts (Table S1).

**Figure 10:**
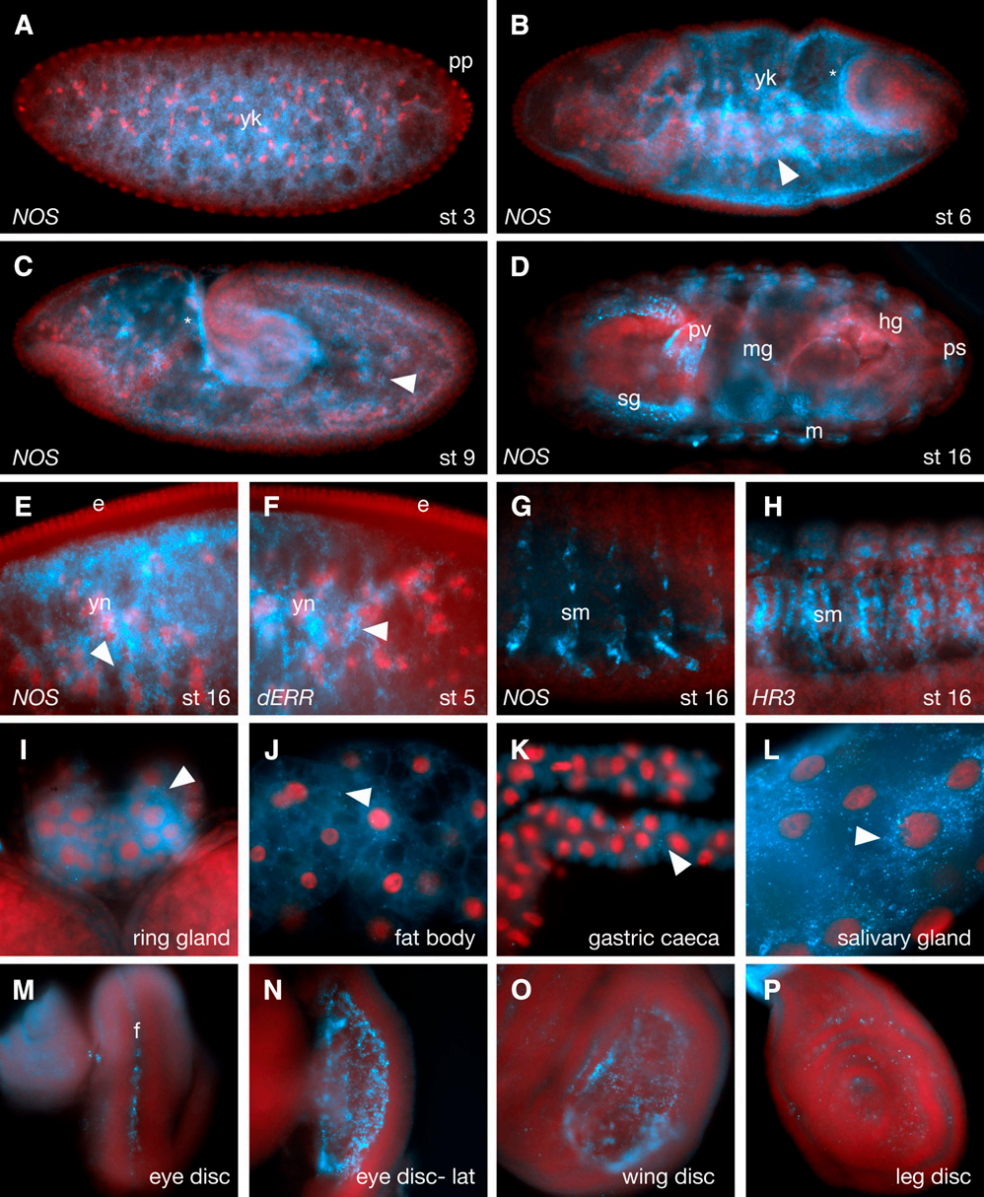
Expression of *NOS*. (A) Early stage embryo showing modest maternal expression with enrichment in the yolk (yk) away from the periphery and pole plasm (pp). (B) During gastrulation zygotic expression leads to basally localized transcripts in the epithelial cell layer (asterisks) and new expression in the yolk (yk) with perinuclear localization (arrowhead). (C) This pattern continues during germband extension (asterisk- basal enrichment, arrowhead- yolk nuclei perinuclear localization). During late embryogenesis (D), *NOS* is expressed in the salivary glands (sg), tracheal system (different focal plane), posterior spiracles (ps), hindgut (hg), midgut (mg) and part of the somatic muscles (sm). (E, F) Greater-magnification images of stage 5 embryos showing similarity between *NOS* and *Drosophila Estrogen-related receptor* (*dERR*) localization patterns with enrichment around yolk nuclei (yn; arrowheads). (G, H) Greater-magnification stage 16 embryos showing expression of *NOS* and *HR3* at the somatic muscles (sm). (I−P) 3rd instar larval expression patterns showing expression in the prothoracic glands (I), fat body (J), portions of the gastric caeca (K), salivary gland (L) and eye (M, dorsal view; N, lateral view), wing (O) and leg (P) imaginal discs. Arrowheads show features of subcellular localization. F, morphogenic furrow.

During early embryonic stages, low levels of maternal *NOS* transcript are observed in the yolk, with restriction from the cortex and pole plasm, much as described for most of the early expressed NR transcripts (Figure 10A). Subsequent zygotic NOS expression closely resembles that of E78, with restriction to basal epithelium and yolk (Figure 10, B and C). At late embryonic stages, *NOS* mRNA is seen in the salivary glands, developing tracheal system, posterior spiracles, apical hindgut, somatic muscles, proventriculus and areas of the head (Figure 10D). It can also be detected at lower levels in the border between the hindgut and midgut, yolk nuclei inside the midgut, and the prothoracic gland (Figure 2, Table S2, and http://fly-fish.ccbr.utoronto.ca).

During larval stages, *NOS* mRNA is seen in the prothoracic gland, proventriculus, some neurons in the brain lobes, eye, wing and leg discs, midgut, gastric caeca, salivary glands, fat body, and Malpighian tubules, as well as scattered puncta in the larval testis (summarized in Figures 2 and 10, I−P; Table S2; and Table S3). Thus, *NOS* is expressed in the majority of tissues that are enriched for NR expression, and, as expected, relates particularly closely to a subset of patterns of *E75*, *HR3*, *HR51*, and others. The morphogenic furrow of the eye disc is an interesting example, with all four genes expressed in, around and/or under the advancing furrow (Figures 8, E and F and 10M and data not shown).

## Discussion

This high-level, comprehensive analysis of all 18 *Drosophila* NR expression patterns will serve as a useful reference for NR functions over the course of development. It also provides a number of inferred relationships, novel insights, and surprises, with numerous implications for their vertebrate homologues as well. Many of the inferred functions compare well to a previous study of NR activities obtained using NR fusion proteins and responsive reporters to identify sites of ligand responsiveness and activity (Palanker *et al.* 2006). Although the results of that study were limited to the nine NRs that function as transcriptional activators, it was the first to emphasize a common involvement in tissues and functions involved in metabolism in insects. One of the major surprises of the current study is the breadth of NRs expressed in these tissues. In several cases, this included all 18 NRs, clearly highlighting the close functional relationships of these genes, as well as the complexity of the processes being regulated.

### Tissues and NRs involved in metabolism

The first tissue to show a clear NR enrichment during development was the maternally provided yolk, which serves as a repository of the biological fuels and building blocks required for embryonic development and hatching. In many cases, these molecules are broken down or released as required from more complex storage or precursor molecules. Ecdysone, for example, which is provided maternally, is not required until mid-embryogenesis. Until that time, it is stored in the yolk linked via phosphate bonds to carrier proteins (Hoffmann and Lagueux 1985; Lagueux *et al.* 1977, 1981; Bownes *et al.* 1988a,b). Its release at mid-embryogenesis leads to EcR activity in the midgut and surrounding amnioserosa, with subsequent downstream NR expression and activities in both tissues (Palanker *et al.* 2006).

Appropriately, the yolk ends up being engulfed by the midgut, which is another common site of NR expression. This ongoing expression in the midgut, and other parts of the gut, is consistent with the roles of NRs in sensing and regulating metabolite and toxin levels in food, and then promoting appropriate local and systemic responses.

In most responsive tissues, the response to ecdysone triggers a transcriptional cascade of NR responses (Ashburner 1972; Fahrbach *et al.* 2012). In the yolk, however, we see that the majority of NR transcripts are already present due to maternal deposition. This, together with the lack of activity detected (E78 excepted) until mid-embryogenesis (Palanker *et al.* 2006), indicates additional posttranscriptional levels of regulation. This could include differential regulation via mRNA localization (see *Subcellular transcript localization* below), translation or, as described earlier for ecdysone, ligand availability. This area will be an interesting one to pursue because similar levels of regulation are likely to be conserved in vertebrates.

After gastrulation, the mesoderm was also a common site of NR expression. This finding seems logical because the mesoderm gives rise to a number of other common NR expressing tissues, including the fat body, gonads, visceral mesoderm, hemocytes, lymph glands, heart, and muscles. The fat body, in particular, was a major site of observed NR expression, with all 18 NR transcripts detected at some point during embryonic and larval development. In addition to storing lipids, the fat body is the primary secretory organ of the larvae, with analogous functions to the human liver and adipose tissue (Charroux and Royet 2010; Kuhnlein 2011). Lipid storage in the fat body peaks as the 3rd instar larva reach their maximum size. Then, upon ecdysone release from the prothoracic gland, and subsequent onset of metamorphosis, the process begins to reverse as fat body cells undergo autophagy, releasing their lipid contents to help fuel the major morphological changes that follow.

The same well-documented cascade of NR functions that has been documented in the salivary glands after their response to ecdysone production (Ashburner 1972; Huet *et al.* 1995; Gonsalves *et al.* 2011), and in the prothoracic gland before ecdysone secretion (Caceres *et al.* 2011), also occurs in the fat body (Antoniewski *et al.* 1994; Wang *et al.* 2010). The coincident expression of all 18 NRs in the fat body at the end of larval development is indicative of a number of other yet to be determined functions at this time.

In the larval prothoracic gland, the production of ecdysone is under neuroendocrine control from insulin-producing cells, and both the circadian and prothoracicotropic hormone−secreting neurons of the brain. Consequently, the production of ecdysone occurs in a relatively circadian fashion towards the end of each instar as the larvae reach maximal size. Again, it makes sense that NRs, which can both sense and regulate lipid and hormone metabolism, are expressed in this tissue, as ecdysone production and metamorphosis should not proceed before sufficient levels of metabolites required to fuel the process are available.

Another endocrine tissue that works in close association with the fat body and prothoracic gland are the oenocytes (Gutierrez *et al.* 2007). These segmentally repeated clusters of endocrine cells at the epidermal surface help keep track of overall lipid content, with appropriate responses to rich and lean diets. Interestingly, lipid content in these cells tends to be reciprocal to that of the fat body, and other tissues in general (Gutierrez *et al.* 2007). NRs detected here during mid- to late embryonic stages include *EcR*, *dHNF4*, and *svp*. Others are likely to be expressed here at other stages and under different conditions.

Another set of organs involved in metabolism and metabolic waste are the Malpighian tubules. Like the human kidneys, these filter good and bad metabolites, as appropriate, as well as controlling systemic levels of hydration. Again, the observation of virtually all NRs showing detectable expression in this organ (*HR38* excepted) attests to the complexity of processes being regulated here. Although fewer NRs were detected here in the embryo, this may be due to the small size of the tubular structures at these stages, or to relative inactivity prior to hatching.

One common site of NR expression without an obvious link to metabolism was the lymph gland. Again, the expression of all 18 NRs just before metamorphosis suggests important and complex activities. Some may be related to the consequences and demands of widespread tissue removal and restructuring about to take place.

### Other common functions suggested by ontological clustering

In an effort to decipher other common functions and links among the NRs, expression and subcellular localization terms were annotated and entered into a functional clustering algorithm (Gene Cluster, Java TreeView). Although the results of such clustering were largely consistent with the observations made above, the large amounts of data from different stages and tissues made the results highly dependent upon the amount of data included and how the terms were weighted. Two representative clusters are shown in Figure S1 and Figure S2. The first one includes terms that relate only to tissue expression (Figure S1), and the second using only subcellular localization terms (Figure S2 and Table S3). Although these relationships are likely to provide new insights into NR regulation, interactions and functions, the data are too diverse and preliminary to discuss succinctly without additional supporting data. Unraveling these relationships further will require additional molecular and genetic approaches that determine which interactions are direct.

### Uniquely expressed NRs

Although all NRs shared at least a few common sites of expression, due most likely to related evolutionary origins and functions, each also displayed unique sites and patterns of expression, such as those seen in the CNS and imaginal discs. Several NRs, however, were expressed in a more unique or restricted fashion. *Tailless*, for example, was the earliest to be expressed zygotically, beginning at stage 3, with its role in anteroposterior patterning. As such, it was the only NR not to be excluded from posterior cells. Notably, some of these *tll*-expressing cells give rise to the pole cells and endoderm. Pole cells are the germ line stem cell precursors, and endoderm cells give rise to the midgut. Interestingly, recent attention has focused on the roles of *tll*, and its vertebrate orthologue *Tlx*, in neural stem cell renewal (Sun *et al.* 2007; Li *et al.* 2008; Park *et al.* 2010; Qu *et al.* 2010). The persistent expression of *tll* observed here in pole cells suggests the possibility of a related stem cell role in the germ line.

The heme-binding hormone NR HR51 is homologous to the vertebrate receptor photoreceptor-specific nuclear receptor. In a comprehensive study of NR expression in mice, photoreceptor-specific nuclear receptor was found to be expressed almost exclusively in the eye (Bookout *et al.* 2006). In *Drosophila*, however, it has been shown to modulate wing expansion, mushroom body development and fertility. Consistently, expression was observed in numerous tissues in addition to the eyes, although many of these were neuronal in nature. Interestingly, as with *E75*, *HR3*, and *NOS*, *HR51* was also expressed in the vicinity of the morphogenic furrow of the eye imaginal disc. However, it did not appear to be expressed within the furrow itself, suggesting a role that is opposing or incremental to those of the other three proteins.

### Subcellular transcript localization

One of the major surprises of this study was the extent of subcellular localization exhibited by NR transcripts (see Table S3 for a list and summary of all terms). Although recent studies have shown that the percentage of mRNAs that show non-random localization is much higher than expected (Lecuyer *et al.* 2007), it is still generally assumed that, given their ability to relocate into the nucleus, transcription factor mRNAs are unlikely to exhibit or require this level of regulation. However, this study suggests that subcellular localization may also be a fairly frequent event for other transcription factor transcripts, providing a unique way to temporally and spatially coordinate the functions of otherwise independently regulated genes (Lecuyer *et al.* 2009).

In the case of the maternally provided NR transcripts, which were the majority, transcripts were consistently depleted from the periphery during the periods of nuclear divisions and cellularization. They were also depleted from pole plasm and pole cells, suggesting either a lack of requirement, or a need to have them inactive in these cells. Strikingly, *E78* transcripts were localized much the same as the maternally provided NR transcripts, with enrichment in the yolk and eventual exclusion from the epidermal cell layer, despite having been expressed by those same epidermal cells. This convergence of localization patterns by maternally and zygotically expressed mechanisms is both interesting and consistent with a common requirement within the yolk plasm, and or nuclei.

At later stages, NR transcripts expressed in the mesoderm and developing gut were consistently enriched toward the side of each cell that faces the yolk. This localization may be related to roles for the encoded proteins in sensing or regulating the nearby gut contents. Within the yolk-filled midgut, transcripts were also seen enriched along filamentous projections. These may provide clues regarding the mechanisms of transcript localization. As discussed earlier, this localization may also be related to roles in the timed metabolism and deployment of maternally deposited lipids and hormones out of the yolk and into adjacent tissues.

In larvae, the large sizes of many polyploid cell types helped to expose other types of subcellular distribution and with better resolution. In many of these cases, transcripts tended to be enriched perinuclearly along what appears to be ER. In some tissues, transcripts also were enriched to one side of the nucleus, suggesting either a polarized export process, or a close proximity to the sites of nascent transcription. This localization may also reflect roles for NR transcripts or proteins in the cytoplasm, or simply a means of making subsequent nuclear protein transport more efficient or controllable. At almost all stages and in most cells, NR transcripts in the cytoplasm tended to accumulate in distinct particles. The number and sizes of these particles varied between NRs and tissue types.

Finally, we also noted that, on occasion, some NR transcripts (*e.g.*, *tll*, *svp*, *dsf*) appeared to be retained within the nucleus. This type of localization has previously been observed with the gap genes *hunchback* and *castor* (Lecuyer *et al.* 2007) and likely represents a novel form of translational control.

### NRs and processes regulated by NO

NOS, and the presence of NO gas, have previously been shown to be involved in ecdysone production and ecdysone-regulated activities via regulation of the NR E75 in the prothoracic gland (Caceres *et al.* 2011). Consistent with these relationships, we found *NOS* to be co-expressed with *E75* (and its heterodimer partner HR3), as well as the downstream target *Ftz-F1*, in numerous other developing tissues. These include fat body, various parts of the gut, trachea, malphigian tubules, salivary glands, CNS, and eye discs, suggesting common roles in the morphogenesis and functions of these tissues. Many of these are consistent with other known or suspected functions of these genes or their human orthologues. For example, expression during tracheal development could control aspects of cuticle production, fluid clearing or oxygen (hypoxia) detection. In the eye discs and parts of the brain such as the optic lobes, expression may help to establish many of the neuronal connections that facilitate light detection and circadian behaviors.

Consistent with previous findings indicating that, like E75, HR51 can also binds heme and NO, we also found a strong correlation between *NOS* and *HR51* expression patterns, both at the cellular and subcellualr level. In fact, clustering analyses consistently placed these two genes as close or closer than *E75* and *NOS*.

### Further studies required

Although this study was relatively extensive and comprehensive, there are many additional details that will need to be addressed prior to obtaining a full understanding of NR functions during development. First, it should be noted that about half of the *Drosophila* NRs encode more than one transcript isoform, with many encoding three or more. The proteins encoded by these transcripts may have significantly divergent functions. The probes used here were designed to recognize all or most isoforms (see Table S1).

We also did not manage to look at earlier larval instar expression patterns, or within pupae and adults. Additional important considerations will be sex-specific and circadian patterns of expression, as well as responses to diet, toxins, injury and other situational stimuli and stresses. Finally, greater-resolution imaging, together with appropriate cellular markers, will be required to definitively identify all sites of cellular and subcellular expression.

## Supplementary Material

Supporting Information

## References

[bib1] Aicart-Ramos C. Valhondo Falcon M. Ortiz de Montellano P. R. Rodriguez-Crespo I., 2012 Covalent attachment of heme to the protein moiety in an insect E75 nitric oxide sensor. Biochemistry 51(37): 7403–7416 22946928 10.1021/bi300848x PMC3448046

[bib2] Anbalagan M. Huderson B. Murphy L. Rowan B. G., 2012 Post-translational modifications of nuclear receptors and human disease. Nucl. Recept. Signal. 10: e001 22438791 10.1621/nrs.10001 PMC3309075

[bib3] Antoniewski C. Laval M. Dahan A. Lepesant J. A., 1994 The ecdysone response enhancer of the Fbp1 gene of Drosophila melanogaster is a direct target for the EcR/USP nuclear receptor. Mol. Cell. Biol. 14(7): 4465–4474 8007953 10.1128/mcb.14.7.4465-4474.1994 PMC358818

[bib4] Ashburner M., 1972 Patterns of puffing activity in the salivary gland chromosomes of Drosophila. VI. Induction by ecdysone in salivary glands *of D. melanogaster* cultured in vitro. Chromosoma 38(3): 255–281 4627363 10.1007/BF00290925

[bib5] Bookout A. L. Jeong Y. Downes M. Yu R. T. Evans R. M., 2006 Anatomical profiling of nuclear receptor expression reveals a hierarchical transcriptional network. Cell 126(4): 789–799 16923397 10.1016/j.cell.2006.06.049 PMC6211849

[bib6] Bownes M. Scott A. Shirras A., 1988 a Dietary components modulate yolk protein gene transcription in *Drosophila melanogaster*. Development 103(1): 119–128 2461849 10.1242/dev.103.1.119

[bib7] Bownes M. Shirras A. Blair M. Collins J. Coulson A., 1988 b Evidence that insect embryogenesis is regulated by ecdysteroids released from yolk proteins. Proc. Natl. Acad. Sci. USA 85(5): 1554–1557 3125550 10.1073/pnas.85.5.1554 PMC279811

[bib8] Caceres L. Necakov A. S. Schwartz C. Kimber S. Roberts I. J., 2011 Nitric oxide coordinates metabolism, growth, and development via the nuclear receptor E75. Genes Dev. 25(14): 1476–1485 21715559 10.1101/gad.2064111 PMC3143938

[bib9] Charroux B. Royet J., 2010 Drosophila immune response: from systemic antimicrobial peptide production in fat body cells to local defense in the intestinal tract. Fly (Austin) 4(1): 40–47 20383054 10.4161/fly.4.1.10810

[bib10] Copeland J. W. Nasiadka A. Dietrich B. H. Krause H. M., 1996 Patterning of the Drosophila embryo by a homeodomain-deleted Ftz polypeptide. Nature 379(6561): 162–165 8538765 10.1038/379162a0

[bib11] Fahrbach S. E. Smagghe G. Velarde R. A., 2012 Insect nuclear receptors. Annu. Rev. Entomol. 57: 83–106 22017307 10.1146/annurev-ento-120710-100607

[bib12] Gollamudi R. Gupta D. Goel S. Mani S., 2008 Novel orphan nuclear receptors-coregulator interactions controlling anti-cancer drug metabolism. Curr. Drug Metab. 9(7): 611–613 18781912 10.2174/138920008785821701

[bib13] Gonsalves S. E. Neal S. J. Kehoe A. S. Westwood J. T., 2011 Genome-wide examination of the transcriptional response to ecdysteroids 20-hydroxyecdysone and ponasterone A in Drosophila melanogaster. BMC Genomics 12: 475 21958154 10.1186/1471-2164-12-475 PMC3228561

[bib14] Guichet A. Copeland J. W. Erdelyi M. Hlousek D. Zavorszky P., 1997 The nuclear receptor homologue Ftz-F1 and the homeodomain protein Ftz are mutually dependent cofactors. Nature 385(6616): 548–552 9020363 10.1038/385548a0

[bib15] Gutierrez E. Wiggins D. Fielding B. Gould A. P., 2007 Specialized hepatocyte-like cells regulate Drosophila lipid metabolism. Nature 445(7125): 275–280 17136098 10.1038/nature05382

[bib40] Hoffmann J. A. Lagueux M., 1985 Endocrine aspects of embryonic development in insects, pp. 435–460 in *Comprehensive Insect Physiology, Biochemistry and Pharmacology*, Vol. 1, edited by G. A. Kerkut and L. I. Gilbert. Pergamon, Oxford, New York.

[bib16] Huet F. Ruiz C. Richards G., 1995 Sequential gene activation by ecdysone in Drosophila melanogaster: the hierarchical equivalence of early and early late genes. Development 121(4): 1195–1204 7743931 10.1242/dev.121.4.1195

[bib17] Jung S.-H. Evans C. J. Uemura C. Banerjee U., 2005 The Drosophila lymph gland as a developmental model of hematopoiesis. Development 132(11): 2521–2533 15857916 10.1242/dev.01837

[bib18] King-Jones K. Thummel C. S., 2005 Nuclear receptors—a perspective from Drosophila. Nat. Rev. Genet. 6(4): 311–323 15803199 10.1038/nrg1581

[bib19] Krause H. M. Klemenz R. Gehring W. J., 1988 Expression, modification, and localization of the fushi tarazu protein in Drosophila embryos. Genes Dev. 2(8): 1021–1036 3049237 10.1101/gad.2.8.1021

[bib20] Krzemien J. Oyallon J. Crozatier M. Vincent A., 2010 Hematopoietic progenitors and hemocyte lineages in the Drosophila lymph gland. Dev. Biol. 346(2): 310–319 20707995 10.1016/j.ydbio.2010.08.003

[bib21] Kuhnlein R. P., 2011 The contribution of the Drosophila model to lipid droplet research. Prog. Lipid Res. 50(4): 348–356 21620889 10.1016/j.plipres.2011.04.001

[bib22] Lagueux M. Hirn M. Hoffmann J. A., 1977 Ecdysone during ovarian development in Locusta migratoria. J. Insect Physiol. 23(1): 109–119 858928 10.1016/0022-1910(77)90116-0

[bib23] Lagueux M. Harry P. Hoffmann J. A., 1981 Ecdysteroids are bound to vitellin in newly laid eggs of Locusta. Mol. Cell. Endocrinol. 24(3): 325–338 7035253 10.1016/0303-7207(81)90007-1

[bib24] Lecuyer E. Parthasarathy N. Krause H. M., 2008 Fluorescent in situ hybridization protocols in Drosophila embryos and tissues. Methods Mol. Biol. 420: 289–302 18641955 10.1007/978-1-59745-583-1_18

[bib25] Lecuyer E. Yoshida H. Krause H. M., 2009 Global implications of mRNA localization pathways in cellular organization. Curr. Opin. Cell Biol. 21(3): 409–415 19249199 10.1016/j.ceb.2009.01.027

[bib26] Lecuyer E. Yoshida H. Parthasarathy N. Alm C. Babak T., 2007 Global analysis of mRNA localization reveals a prominent role in organizing cellular architecture and function. Cell 131(1): 174–187 17923096 10.1016/j.cell.2007.08.003

[bib27] Li W. Sun G. Yang S. Qu Q. Nakashima K., 2008 Nuclear receptor TLX regulates cell cycle progression in neural stem cells of the developing brain. Mol. Endocrinol. 22(1): 56–64 17901127 10.1210/me.2007-0290 PMC2194628

[bib28] Palanker L. Necakov A. S. Sampson H. M. Ni R. Hu C., 2006 Dynamic regulation of Drosophila nuclear receptor activity in vivo. Development 133(18): 3549–3562 16914501 10.1242/dev.02512 PMC2100403

[bib29] Pardee K. Necakov A. S. Krause H., 2011 Nuclear receptors: small molecule sensors that coordinate growth, metabolism and reproduction. Subcell. Biochem. 52: 123–153 21557081 10.1007/978-90-481-9069-0_6

[bib30] Park H. J. Kim J. K. Jeon H. M. Oh S. Y. Kim S. H., 2010 The neural stem cell fate determinant TLX promotes tumorigenesis and genesis of cells resembling glioma stem cells. Mol. Cells 30(5): 403–408 20814749 10.1007/s10059-010-0122-z

[bib31] Qu Q. Sun G. Li W. Yang S. Ye P., 2010 Orphan nuclear receptor TLX activates Wnt/beta-catenin signalling to stimulate neural stem cell proliferation and self-renewal. Nat. Cell Biol. 12(1): 31-40; sup pp 1-9 20010817 10.1038/ncb2001 PMC2880892

[bib32] Reinking J. Lam M. M. Pardee K. Sampson H. M. Liu S., 2005 The Drosophila nuclear receptor e75 contains heme and is gas responsive. Cell 122(2): 195–207 16051145 10.1016/j.cell.2005.07.005

[bib33] Roehrborn G., 1961 Drosophila tumors and the structure of larval lymph glands. Experientia 17: 507–509 14493256 10.1007/BF02158625

[bib34] Schwartz C. J. Sampson H. M. Hlousek D. Percival-Smith A. Copeland J. W., 2001 FTZ-Factor1 and Fushi tarazu interact via conserved nuclear receptor and coactivator motifs. EMBO J. 20(3): 510–519 11157757 10.1093/emboj/20.3.510 PMC133472

[bib35] Sun G. Yu R. T. Evans R. M. Shi Y., 2007 Orphan nuclear receptor TLX recruits histone deacetylases to repress transcription and regulate neural stem cell proliferation. Proc. Natl. Acad. Sci. USA 104(39): 15282–15287 17873065 10.1073/pnas.0704089104 PMC2000559

[bib36] Thummel C. S., 2001 Molecular mechanisms of developmental timing in C. elegans and Drosophila. Dev. Cell 1(4): 453–465 11703937 10.1016/s1534-5807(01)00060-0

[bib37] Vacca M. Degirolamo C. Mariani-Costantini R. Palasciano G. Moschetta A., 2011 Lipid-sensing nuclear receptors in the pathophysiology and treatment of the metabolic syndrome. Wiley Interdiscip. Rev. Syst. Biol. Med. 3(5): 562−587 21755605 10.1002/wsbm.137

[bib38] Wang S. Liu S. Liu H. Wang J. Zhou S., 2010 20-hydroxyecdysone reduces insect food consumption resulting in fat body lipolysis during molting and pupation. J. Mol. Cell Biol. 2(3): 128–138 20430856 10.1093/jmcb/mjq006

[bib39] Wilk R. Murthy S. U. M. Yan H. Krause H. M., 2010 In Situ Hybridization: Fruit Fly Embryos and Tissues. Current Protocols Essential Laboratory Techniques. John Wiley & Songs, Inc, New York

