# Supplementary material for: Spatial Profiling of Nuclear Receptor Transcription Patterns over the Course of Drosophila Development
Source: G3 (Bethesda). 2013 Jul 1;3(7):1177–89. doi: 10.1534/g3.113.006023 (PMC3704245; doi:10.1534/g3.113.006023)
Supplement: Supporting Information [file supp_3_7_1177__index.html]

Spatial Profiling of Nuclear Receptor Transcription Patterns over the Course of Drosophila Development — Supporting Information 

# Spatial Profiling of Nuclear Receptor Transcription Patterns over the Course of *Drosophila* Development

## Supporting Information for Wilk, Hu, and Krause, 2013

**Files in this Data Supplement:**

- Supporting Information - Figures S1-S2 and Tables S1-S3 (PDF, 1 MB)
- Figure S1 - Expression pattern relationships (PDF, 666 KB)
- Figure S2 - Subcellular localization relationships (PDF, 591 KB)
- Table S1 - *Drosophila* nuclear receptor and *NOS* isoforms and probes (PDF, 171 KB)
- Table S2 - Summary of FISH expression data (PDF, 142 KB)
- Table S3 - Summary of FISH subcellular patterns (PDF, 143 KB)
