# Supplementary material for: Spatial Profiling of Nuclear Receptor Transcription Patterns over the Course of Drosophila Development
Source: G3 (Bethesda). 2013 Jul 1;3(7):1177–89. doi: 10.1534/g3.113.006023 (PMC3704245; doi:10.1534/g3.113.006023)
Supplement: Supporting Information [file supp_g3.113.006023_006023SI.pdf]

G3: Genes | Genomes | Genetics

Spatial profiling of nuclear receptor transcription patterns over the course of *Drosophila* development

Ronit Wilk, Jack Hu and Henry M. Krause

DOI: 10.1534/g3.113.006023

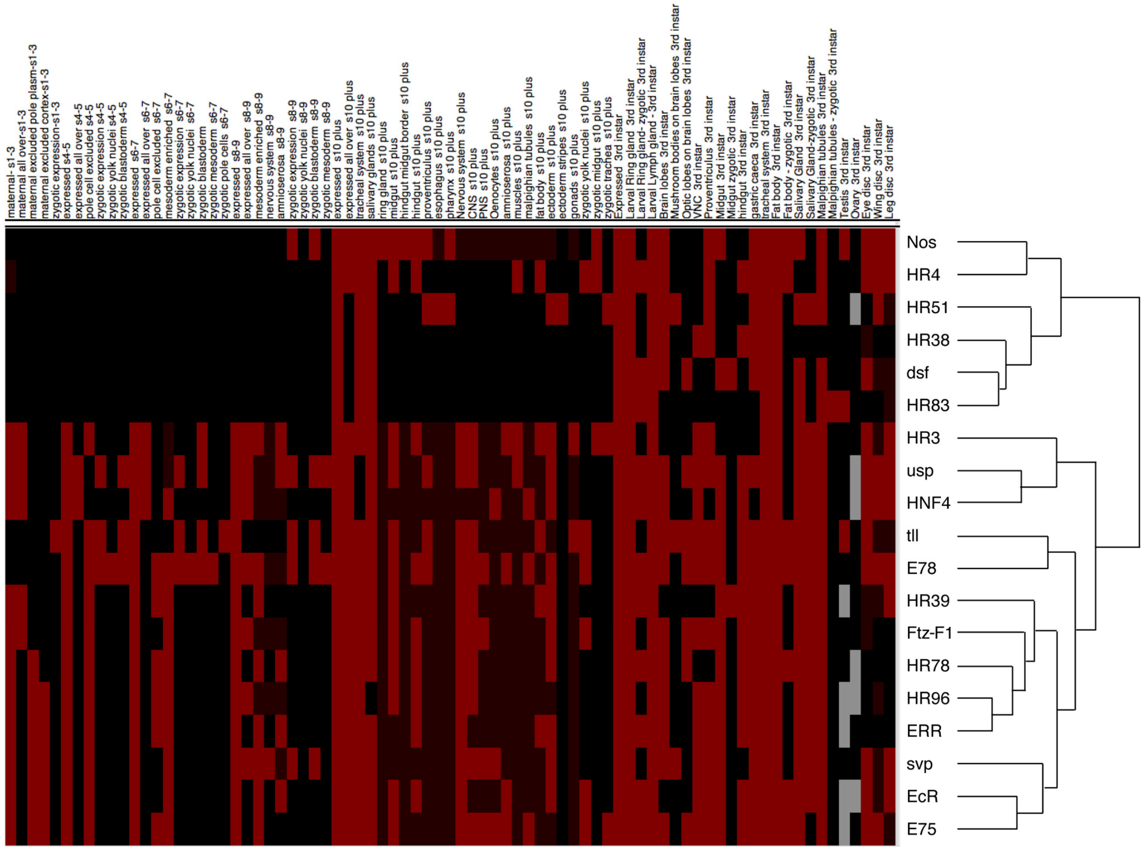

Figure S1 Expression pattern relationships. Cluster comparisons between all 18 nuclear receptors and NOS tissue expression patterns was performed using 'Gene Cluster' and 'Java TreeView' software. Gene identities are listed to the left of each row, and terms for the tissues and stages analyzed above each column. Bright red = strong expression; dark red = lower expression; black = no expression; grey = no data. The tree on the right shows grouping by overall similarity.

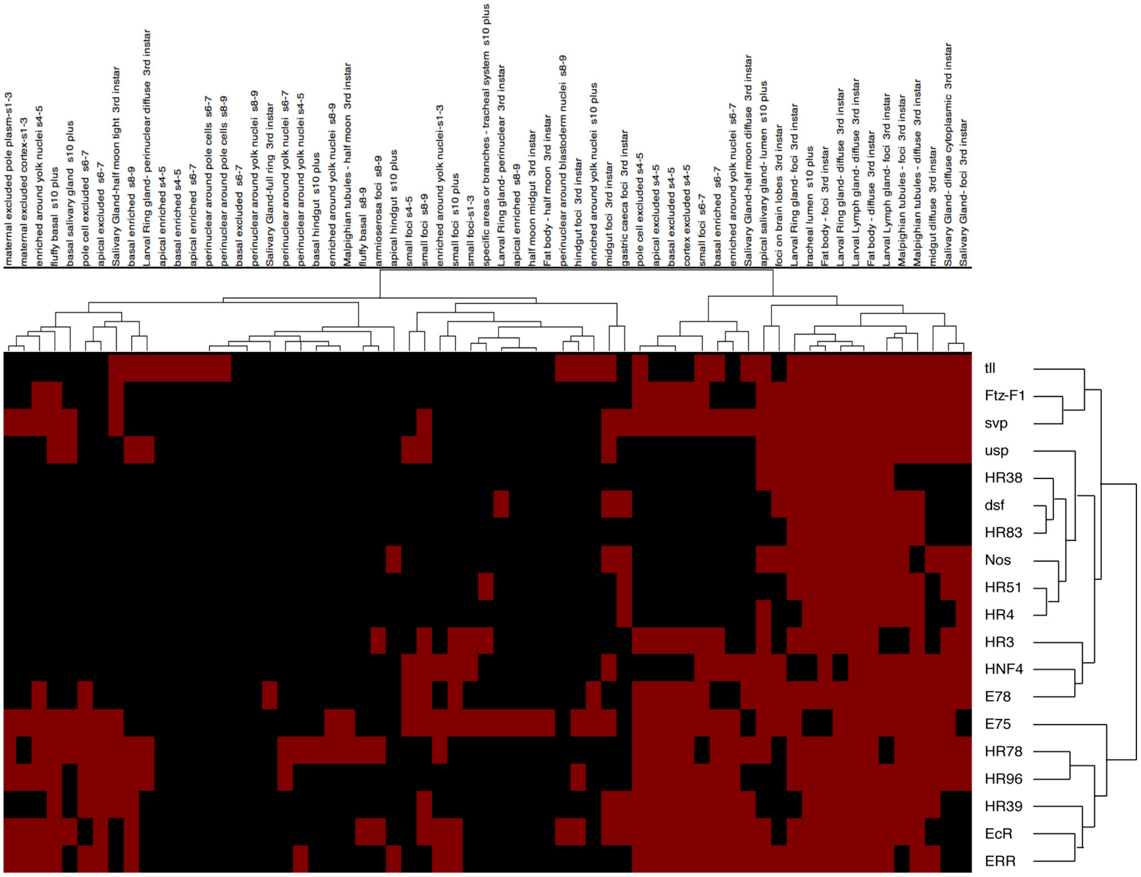

Figure S2 Subcellular localization relationships Cluster comparisons between all 18 nuclear receptors and *NOS* subcellular localization patterns was obtained using 'Gene Cluster' and 'Java TreeView' software. NR gene identities are listed to the right of each row, and terms for subcellular distributions and stages analyzed are listed above each column. red = expression; black = no expression;. The trees on the right and the top show groupings by overall similarity.

Table S1 ***Drosophila*** **nuclear receptor and *NOS* isoforms and probes.** CG numbers, number of splice forms, and oligos used for probe template production are provided for each of the fly NR genes and *NOS*.

Gene name **(Abbreviation)**	**CG number**	**Number of isoforms**	**Probe name**	**Oligos used (if home made)**	**It hybridizes to (size in base pairs)**
Ecdysone receptor (EcR)	CG1765	6	LD26915	NA	Multiple exons- including some 3'UTR. Covers all isoforms (1896)
ultraspiracle (usp)	CG4380	1	LD09973	NA	The entire transcript (2481)
Hormone receptor-like in 46 (Hr46 or HR3)	CG3318 3	6	GH09429	NA	Multiple exons and UTRs. Covers all isoforms (3827)
			GH21112	NA	Multiple exons including 3'UTR. Covers all isoforms (4248)
Hr4 (HR4)	CG4393 4 CG1690 2	9	HM-DHR4	up: 5'..GACATG AAGCCCAT GTTCCT down*: 5'..ATTAGC GTGGTCAA GCAGGT	Part of last exon. Covers all isoforms and CG42527 (830)
Ecdysone-induced protein 75B (E75)	CG8127	6	GM04985	NA	Multiple exons including part of 3'UTR. Covers of all isoforms (3107)
Ftz transcription factor 1 (Ftz-F1)	CG4059	3	LD34889	NA	Multiple exons and part of 3'UTR. Covers all isoforms (2270)
			LD11885	NA	First exon. Covers RB isoform (842)
Hormone receptor-like in 39 (HR39)	CG8676	4	LD45021	NA	Almost the entire transcript, Covers all isoforms (2999)
Hormone-receptor-like in 78 (HR78)	CG7199	6	GH08073	NA	The entire transcript. Covers all isoforms (2283)
Hormone receptor-like in 96 (HR96)	CG1178 3	1	GH14435	NA	The entire transcript (2834)
estrogen-related receptor (dERR)	CG7404	2	GH28308	NA	The entire transcript. Covers both isoforms (2125)
Hepatocyte nuclear factor 4 (dHNF4)	CG9310	4	RE09535	NA	The entire transcript. Covers all isoforms (3333)
seven up (svp)	CG1150 2	3	AT29920	NA	The entire transcript. Covers RA and RB isoforms (2085)
			GH08189	NA	3' UTR. Covers RC and RB isoforms (2023)
Ecdysone-induced protein 78C (E78)	CG1802 3	4	HM-E78-Ex10	up: 5'..CTTGCT GAACTTTG CCAACA down*: 5'..GCTCGC TCTTCAGT CGAGTT	Last exon, including part of 3'UTR. Covers all isoforms (764)

dissatisfaction (dsf)	CG9019	1	HM-dsf-Ex4	up: 5'..ACTCCA TCCAGTCC ATCTCG<br><br>down*: 5'..AGCAAA TGCTGGTC GTTCTT	Part of exon 4 (637)
talilles (tll)	CG1378	1	HM-tll-Ex3	up: 5'..AGGGAC TCTGTGTG GTGGAC<br><br>down*: 5'..TTGAGA CCTTGTGC ATCAGC	Most of exon 3 (1041)
Hormone receptor 51 (HR51)	CG1680 1	1	HM-DHR51-Ex6	up: 5'..ACCACC TCACCTCC TCAATG<br><br>down*: 5'..AAGAGC TGACGGAG GAGACA	Most of exon 6 (249)
Hormone receptor-like in 38 (HR38)	CG1864	3	HM-DHR38	up: 5'..CGACCA CGTCACCT ACAATG<br><br>down*: 5'..TAGTGC CGAATGTT GAGCAG	Last exon and part of 3'UTR. Covers of all isoforms (644)
Hormone receptor 83 (HR83)	CG1029 6	1	HM-DHR83-Ex1	up: 5'..GGTGCT CCTGCTTT TTCAAG<br><br>down*: 5'..GCACAG GATTAGGG ACTCCA	Most of exon 1 (542)
Nitric oxide synthase (Nos)	CG6713	10	HM-NOS-Ex16#	up: 5'..GAACGA TTCGCTGA CTGTGA<br><br>down*: 5'..CTTCGG AATGGCCT GCTCC	Part of exon 16. Covers 8/10 isoforms (not Nos-RJ nor Nos-RG) (1078)

*-The sequence: 'GTAATACGACTCACTATAGGGAGACCAC' was added before each reverse oligo sequence to add a T7 polymerase binding site.

#- For the HM-NOS-E16 probe, PCR was first performed on genomic DNA with the following oligos: up-TCGGCCTATCCAAATTTCTG and down- ACTCCTCGGTCCAAACACAC (1871 bp). The product was then amplified using the oligos indicated in the table to generate the 1078 bp template.

Table S2 Summary of FISH expression data.

Gene	Stage	Yolk	Gut	Hindgut	Foregut	Trachea	Post. spiracles	Fat Body	Ectoderm	PNS	CNS	Muscles	Oenocytes	Amnioserosa	Salivary glands	Malpighian tubules	Lymph gland	Ring gland	Brain lobes
EcR *	**E1-5**	+	NA	NA	NA	NA	NA	NA	NA	NA	NA	NA	NA	NA	NA	NA	NA	NA	NA
	**E6-10**	+		NA	NA	NA	NA	NA				NA		+		NA	NA	NA	NA
	**E11-14**		+	+	NA		+			+/-	+/-			+	+	NA	NA	NA	NA
	**E15-17**		+	+	+	+	+		+		+/-			NA	+		NA	NA	NA
	**L3**	NA	+	+	+	+	NA	+	NA	NA	+	NA	NA	NA	+	+	+	+	+
Usp ~*	**E1-5**	+	NA	NA	NA	NA	NA	NA	NA	NA	NA	NA	NA	NA	NA	NA	NA	NA	NA
	**E6-10**	+	+	NA	NA	NA	NA	NA	+/-		+/-	NA		+		NA	NA	NA	NA
	**E11-14**	+	+		NA				+		+			+/-	+	NA	NA	NA	NA
	**E15-17**	+	+		+				+		+	+		NA	+	+/-	NA	NA	NA
	**L3**	NA	+	+	+	+	NA	+	NA	NA	+	NA	NA	NA	+	+	+	+	+
HR3	**E1-5**	+	NA	NA	NA	NA	NA	NA	NA	NA	NA	NA	NA	NA	NA	NA	NA	NA	NA
	**E6-10**	+	+	NA	NA	NA	NA	NA	+			NA		+		NA	NA	NA	NA
	**E11-14**	+	+	+	NA	+	+	+	+			+		+	+	NA	NA	NA	NA
	**E15-17**	+	+	+	+	+	+	+	+	+	+	+		NA	+		NA	NA	NA
	**L3**	NA	+	+	+	+	NA	+	NA	NA	+	NA	NA	NA	+	+	+	+	+
HR4	**E1-5**	+	NA	NA	NA	NA	NA	NA	NA	NA	NA	NA	NA	NA	NA	NA	NA	NA	NA
	**E6-10**			NA	NA	NA	NA	NA				NA				NA	NA	NA	NA
	**E11-14**	+	+	+	NA							+			+	NA	NA	NA	NA
	**E15-17**	+	+	+	+	+	+	+				+		NA	+		NA	NA	NA
	**L3**	NA				+	NA	+	NA	NA		NA	NA	NA	+	+	+	+	+
E75	**E1-5**	+	NA	NA	NA	NA	NA	NA	NA	NA	NA	NA	NA	NA	NA	NA	NA	NA	NA
	**E6-10**	+		NA	NA	NA	NA	NA				NA				NA	NA	NA	NA
	**E11-14**	+	+		NA	+			+	+				+	+	NA	NA	NA	NA
	**E15-17**	+	+		+	+	+/-			+	+		+	NA	+	+	NA	NA	NA
	**L3**	NA	+	+	+	+	NA	+	NA	NA	+	NA	NA	NA	+	+	+	+	+
Ftz-F1 *	**E1-5**	+	NA	NA	NA	NA	NA	NA	NA	NA	NA	NA	NA	NA	NA	NA	NA	NA	NA
	**E6-10**	+		NA	NA	NA	NA	NA				NA				NA	NA	NA	NA
	**E11-14**		+	+	NA	+			+	+/-	+				+	NA	NA	NA	NA
	**E15-17**	+	+		+	+	+		+	+/-	+			NA	+		NA	NA	NA
	**L3**	NA	+	+		+	NA	+	NA	NA	+	NA	NA	NA	+	+	+	+	+
HR39 *	**E1-5**	+	NA	NA	NA	NA	NA	NA	NA	NA	NA	NA	NA	NA	NA	NA	NA	NA	NA
	**E6-10**	+		NA	NA	NA	NA	NA				NA		+		NA	NA	NA	NA
	**E11-14**	+	+		NA			+	+							NA	NA	NA	NA
	**E15-17**		+		+	+	+	+	+		+			NA	+		NA	NA	NA
	**L3**	NA	+	+		+	NA	+	NA	NA		NA	NA	NA	+	+	+	+	
R78	**E1-5**	+	NA	NA	NA	NA	NA	NA	NA	NA	NA	NA	NA	NA	NA	NA	NA	NA	NA

	**E6-10**	+		NA	NA	NA	NA	NA				NA		+		NA	NA	NA	NA
	**E11-14**		+	+	NA	+			+		+				+	NA	NA	NA	NA
	**E15-17**		+	+	+	+		+/-	+		+			NA	+		NA		NA
	**L3**	NA	+	+		+	NA	+	NA	NA		NA	NA	NA	+	+	+	+	+
HR96 *	**E1-5**	+	NA	NA	NA	NA	NA	NA	NA	NA	NA	NA	NA	NA	NA	NA	NA	NA	NA
	**E6-10**	+		NA	NA	NA	NA	NA	+			NA				NA	NA	NA	NA
	**E11-14**		+	+	NA				+		+	+				NA	NA	NA	NA
	**E15-17**		+	+	+	+	+				+			NA			NA	NA	NA
	**L3**	NA	+	+		+	NA	+	NA	NA	+	NA	NA	NA	+	+	+	+	+
dERR *	**E1-5**	+	NA	NA	NA	NA	NA	NA	NA	NA	NA	NA	NA	NA	NA	NA	NA	NA	NA
	**E6-10**	+		NA	NA	NA	NA	NA				NA		+		NA	NA	NA	NA
	**E11-14**		+/-		NA						+			+	+	NA	NA	NA	NA
	**E15-17**			+	+	+	+	+/-	+		+	+		NA	+		NA	NA	NA
	**L3**	NA	+	+	+	+	NA	+	NA	NA	+	NA	NA	NA	+	+	+	+	+
dHNF4 *	**E1-5**	+	NA	NA	NA	NA	NA	NA	NA	NA	NA	NA	NA	NA	NA	NA	NA	NA	NA
	**E6-10**	+		NA	NA	NA	NA	NA	+			NA		+		NA	NA	NA	NA
	**E11-14**		+/-		NA				+				+		+	NA	NA	NA	NA
	**E15-17**		+/-		+				+		+		+	NA	+	+	NA	NA	NA
	**L3**	NA	+	+		+	NA	+	NA	NA	+	NA	NA	NA	+	+	+	+/-	+
Svp *	**E1-5**	+	NA	NA	NA	NA	NA	NA	NA	NA	NA	NA	NA	NA	NA	NA	NA	NA	NA
	**E6-10**	+		NA	NA	NA	NA	NA		+<br>@	+<br>@	NA			+	NA	NA	NA	NA
	**E11-14**		+/-		NA	+	+			+<br>@	+<br>@		+<br>@		+	NA	NA	NA	NA
	**E15-17**				+	+	+	+	+	+<br>@	+<br>@		+<br>@	NA	+ $		NA	NA	NA
	**L3**	NA	+		+	+	NA	+	NA	NA	+<br>@	NA	NA	NA	+	+	+	+	+
E78	**E1-5**	+<br>S5	NA	NA	NA	NA	NA	NA	NA	NA	NA	NA	NA	NA	NA	NA	NA	NA	NA
	**E6-10**	+	+	NA	NA	NA	NA	NA	+			NA		+		NA	NA	NA	NA
	**E11-14**	+	+		NA		+		+	+	+			+	+	NA	NA	NA	NA
	**E15-17**				+	+	+		+					NA	+	+/-	NA	NA	NA
	**L3**	NA	+	+	+	+	NA	+	NA	NA	+	NA	NA	NA	+	+	+	+	+
dsf	**E1-5**		NA	NA	NA	NA	NA	NA	NA	NA	NA	NA	NA	NA	NA	NA	NA	NA	NA
	**E6-10**			NA	NA	NA	NA	NA				NA				NA	NA	NA	NA
	**E11-14**				NA										+	NA	NA	NA	NA
	**E15-17**					+/-	+							NA	+		NA	NA	NA
	**L3**	NA	+			+/-	NA	+	NA	NA		NA	NA	NA	+	+	+	+	+
tll	**E1-5**	+/-<br>%	NA	NA	NA	NA	NA	NA	NA	NA	NA	NA	NA	NA	NA	NA	NA	NA	NA
	**E6-10**	+/-<br>%		NA	NA	NA	NA	NA				NA				NA	NA	NA	NA
	**E11-14**	+	+/-		NA						+				+	NA	NA	NA	NA
	**E15-17**	+	+/-		+	+			+		+			NA	+		NA	NA	NA
	**L3**	NA	+	+	+	+	NA	+	NA	NA	+	NA	NA	NA	+	+	+	+	+

HR51	**E1-5**		NA	NA	NA	NA	NA	NA	NA	NA	NA	NA	NA	NA	NA	NA	NA	NA	NA
	**E6-10**			NA	NA	NA	NA	NA				NA				NA	NA	NA	NA
	**E11-14**				NA	+	+ (^)		+ (#)	+						NA	NA	NA	NA
	**E15-17**				+	+	+ (^)		+ (#)					NA	+		NA	NA	NA
	**L3**	NA				+/-	NA	+	NA	NA		NA	NA	NA	+	+	+	+	+
HR38	**E1-5**		NA	NA	NA	NA	NA	NA	NA	NA	NA	NA	NA	NA	NA	NA	NA	NA	NA
	**E6-10**			NA	NA	NA	NA	NA				NA				NA	NA	NA	NA
	**E11-14**				NA											NA	NA	NA	NA
	**E15-17**					+	+		+/-					NA	+		NA	NA	NA
	**L3**	NA				+	NA	+	NA	NA	+	NA	NA	NA			+	+	
HR83	**E1-5**		NA	NA	NA	NA	NA	NA	NA	NA	NA	NA	NA	NA	NA	NA	NA	NA	NA
	**E6-10**			NA	NA	NA	NA	NA				NA				NA	NA	NA	NA
	**E11-14**				NA										+	NA	NA	NA	NA
	**E15-17**					+								NA	+		NA	NA	NA
	**L3**	NA				+/-	NA	+	NA	NA		NA	NA	NA	+	+	+	+	
Nos	**E1-5**	+/-	NA	NA	NA	NA	NA	NA	NA	NA	NA	NA	NA	NA	NA	NA	NA	NA	NA
	**E6-10**	+	+	NA	NA	NA	NA	NA				NA				NA	NA	NA	NA
	**E11-14**	+	+	+	NA											NA	NA	NA	NA
	**E15-17**	+	+	+	+	+	+	+			+	+		NA	+		NA	NA	NA
	**L3**	NA	+		+	+	NA	+	NA	NA		NA	NA	NA	+	+	+	+	+

+: expressed, NA: not applicable, *: "fluffy" basal localization, ^: posterior spiracle outer layer, #: stripes, s5: stage 5 only, %: *tll* is zygotically expressed at this stage, some transcript ends up at the yolk. ~: low ubiquitous expression, @: specific to *svp*-AT29920 probe, $: specific to *svp*-GH0819 probe. Shaded grey: matches spatial activities noted in Palanker *et al* 2006.

Table S3 Summary of FISH subcellular patterns. A summary of all the subcellular RNA patterns found in this study. The developmental stages are shown at the left of the Table. +: pattern present, -: pattern absent.

Stage	Term	*EcR*	*usp*	*HR3*	*HR4*	*E75*	*Ftz-F1*	*HR39*	*HR78*	*HR96*	*dERR*	*dHNF4*	*svp*	*E78*	*dsf*	*tll*	*HR51*	*HR38*	*HR83*	*Nos*
Embryo 1-3	Pole plasm excluded	+	-	-	-	+	-	-	+	+	+	-	+	-	-	-	-	-	-	-
	Cortex excluded	+	-	-	-	+	-	-	-	+	+	-	+	-	-	-	-	-	-	-
	Small foci	-	-	+	-	+	-	-	-	-	-	+	-	-	-	-	-	-	-	-
	Enriched around yolk nuclei	+	-	-	-	+	-	-	+	-	+	+	-	-	-	-	-	-	-	-
Embryo 4-5	Pole cell excluded	+	-	+	-	+	+	+	+	+	+	-	+	+	-	+	-	-	-	-
	Apical excluded	+	-	+	-	+	+	+	+	+	+	-	+	+	-	-	-	-	-	-
	Basal excluded	+	-	+	-	+	+	+	+	+	+	-	+	+	-	-	-	-	-	-
	Cortex excluded	+	-	+	-	+	+	+	+	+	+	-	+	+	-	-	-	-	-	-
	Apical enriched	-	-	-	-	-	-	-	-	-	-	-	-	-	-	+	-	-	-	-
	Basal enriched	-	-	-	-	-	-	-	-	-	-	-	-	-	-	+	-	-	-	-
	Enriched around yolk nuclei	+	-	-	-	+	+	-	+	+	+	-	+	+	-	-	-	-	-	+
	Perinuclear- yolk nuclei	-	-	-	-	-	-	-	+	-	+	-	-	-	-	-	-	-	-	-
	Small foci	-	+	-	-	+	-	-	-	-	-	+	-	+	-	-	-	-	-	-
Embryo 6-7	Pole cell excluded	-	-	-	-	+	-	+	+	+	+	-	-	+	-	-	-	-	-	-
	Apical excluded	+	-	-	-	+	-	+	+	+	+	-	-	-	-	-	-	-	-	-
	Basal excluded	-	-	-	-	-	-	-	-	-	-	-	-	-	-	-	-	-	-	-
	Apical enriched	-	-	-	-	-	-	-	-	-	-	-	-	-	-	+	-	-	-	-
	Basal enriched	+	-	+	-	+	-	+	+	+	+	+	+	-	-	+	-	-	-	-
	Enriched around yolk nuclei	+	-	-	-	+	-	+	+	+	+	+	+	-	-	-	-	-	-	+
	Perinuclear- yolk nuclei	-	-	-	-	-	-	-	+	+	-	-	-	-	-	-	-	-	-	-
	Perinuclear- pole cells	-	-	-	-	-	-	-	-	-	-	-	-	-	-	+	-	-	-	-
	Small foci	+	-	+	-	+	+	+	-	+	+	+	+	+	-	+	-	-	-	-
Embryo 8-9	Apical enriched	-	-	-	-	+	-	-	-	-	-	-	-	-	-	-	-	-	-	-
	Basal enriched	+	+	-	-	-	-	+	+	+	+	-	-	-	-	+	-	-	-	+
	Fluffy basal	+	-	-	-	-	-	-	+	-	-	-	-	-	-	-	-	-	-	-
	Enriched around yolk nuclei	-	-	-	-	+	-	-	+	-	-	-	-	-	-	-	-	-	-	+
	Perinuclear- yolk nuclei	-	-	-	-	-	-	-	-	-	-	-	-	-	-	-	-	-	-	+/-
	Perinuclear - blastoderm nuclei	+	-	-	-	-	-	-	-	-	-	-	-	-	-	+	-	-	-	+
	Perinuclear- pole cells	-	-	-	-	-	-	-	-	-	-	-	-	-	-	+	-	-	-	-
	Small foci	+	+	+	-	+	-	+	-	-	-	+	+	+	-	-	-	-	-	-
	Amnioserosa - foci	+	-	+	-	-	-	-	+	-	-	-	-	-	-	-	-	-	-	-
Embryo 10-17	Tracheal lumen*	+	+	+	+	-	+	+	+	+	+	-	+	+	+	+	+	+	+	+
	Tracheal system - specific area	-	-	+	-	+	-	-	-	-	-	-	-	-	-	-	+	-	-	-
	Salivary gland - basal	+	+	-	-	+	-	-	+	-	-	-	+	-	-	-	-	-	-	-
	Salivary gland - apical (lumen)*	+	+	+	+	+	+	-	+	-	+	+	+	+	-	+	-	+	-	+
	Hindgut - apical	-	-	-	-	-	-	-	-	-	+	-	-	-	-	-	-	-	-	+

	Hindgut - basal	-	-	-	-	-	-	-	+	-	-	-	-	-	-	-	-	-	-	-
	Enriched around yolk nuclei	-	-	-	-	+	-	-	-	-	-	-	-	+	-	+	-	-	-	-
	Fluffy basal	+	+	-	-	+	+	+	+	+	+	-	+	-	-	-	-	-	-	-
	Small foci	+	-	+	-	+	-	-	-	-	+	+	-	+	-	-	-	-	-	-
Larva 3rd instar	Ring gland - foci	+	+	+	-	+	+	-	+	+	+	-	+	+	+	+	+	+	+	+
	Ring gland - perinuclear	-	-	-	-	+	-	-	-	-	-	-	-	-	+	-	-	-	-	-
	Ring gland - perinuclear diffuse	-	+	-	-	-	-	-	+	+	-	-	-	-	-	+	-	-	-	-
	Brain lobe - foci	-	+	-	-	+	+	-	-	-	+	+	+	+	+	-	-	+	-	+
	Midgut - foci	+	+	-	-	+	-	+	-	-	-	+	+	-	+	+	-	-	-	+
	Midgut - perinuclear half moon	-	-	-	-	+	-	-	-	-	-	-	-	-	-	-	-	-	-	-
	Hindgut - foci	+	-	-	-	+	-	-	-	+	-	-	-	-	-	+	-	-	-	-
	Gastric caeca - foci	+	-	-	+	-	-	+	-	-	-	-	+	-	+	-	+	-	-	+
	Fat body - foci	+	+	+	+	-	+	+	+	+	+	+	+	+	+	+	+	+	+	+
	Fat body - perinuclear half moon	-	-	-	-	+	-	-	-	-	-	-	-	-	-	-	-	-	-	-
	Salivary gland - foci	-	+	+	+	-	+	-	-	+	-	+	+	+	-	+	+	-	-	+
	Salivary gland - perinuclear half moon tight	-	-	-	-	+	+	+	+	+	-	-	+	-	-	+	-	-	-	-
	Salivary gland - perinuclear half moon loose	+	-	-	-	-	-	+	+	-	+	+	+	+	-	+	-	-	-	-
	Salivary gland - perinuclear full	-	-	-	-	-	-	-	-	-	-	-	-	+	-	-	-	-	-	-
	Malpighian tubule - foci	+	+	-	+	+	+	+	+	+	-	+	+	+	+	+	+	-	+	+
	Malpighian tubule - perinuclear half moon	-	-	-	-	+	-	-	+	-	-	-	-	-	-	-	-	-	-	-
