# Supplementary material for: Spatial Profiling of Nuclear Receptor Transcription Patterns over the Course of Drosophila Development
Source: G3 (Bethesda). 2013 Jul 1;3(7):1177–89. doi: 10.1534/g3.113.006023 (PMC3704245; doi:10.1534/g3.113.006023)
Supplement: Supporting Information [file supp_g3.113.006023_FigureS2.pdf]

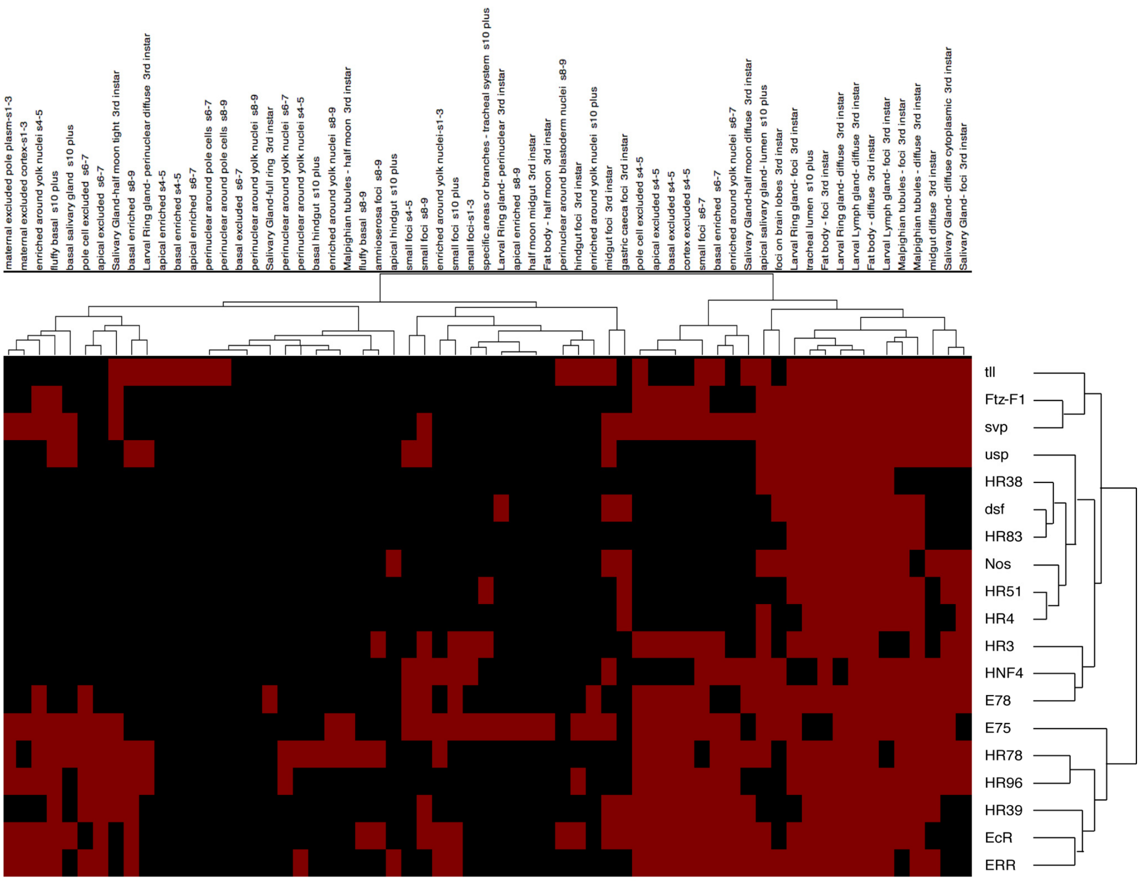

Figure S2 Subcellular localization relationships Cluster comparisons between all 18 nuclear receptors and *NOS* subcellular localization patterns was obtained using 'Gene Cluster' and 'Java TreeView' software. NR gene identities are listed to the right of each row, and terms for subcellular distributions and stages analyzed are listed above each column. red = expression; black = no expression;. The trees on the right and the top show groupings by overall similarity.
