# Supplementary material for: Spatial Profiling of Nuclear Receptor Transcription Patterns over the Course of Drosophila Development
Source: G3 (Bethesda). 2013 Jul 1;3(7):1177–89. doi: 10.1534/g3.113.006023 (PMC3704245; doi:10.1534/g3.113.006023)
Supplement: Supporting Information [file supp_g3.113.006023_TableS1.pdf]

Table S1 ***Drosophila* nuclear receptor and *NOS* isoforms and probes.** CG numbers, number of splice forms, and oligos used for probe template production are provided for each of the fly NR genes and *NOS*.

***Gene name* (Abbreviation)**	**CG number**	**Number of isoforms**	**Probe name**	**Oligos used (if home made)**	**It hybridizes to (size in base pairs)**
Ecdysone receptor (EcR)	CG1765	6	LD26915	NA	Multiple exons- including some 3'UTR. Covers all isoforms (1896)
ultraspiracle (usp)	CG4380	1	LD09973	NA	The entire transcript (2481)
Hormone receptor-like in 46 (Hr46 or HR3)	CG3318 3	6	GH09429	NA	Multiple exons and UTRs. Covers all isoforms (3827)
			GH21112	NA	Multiple exons including 3'UTR. Covers all isoforms (4248)
Hr4 (HR4)	CG4393 4 CG1690 2	9	HM-DHR4	up: 5'..GACATG AAGCCCAT GTTCCT down*: 5'..ATTAGC GTGGTCAA GCAGGT	Part of last exon. Covers all isoforms and CG42527 (830)
Ecdysone-induced protein 75B (E75)	CG8127	6	GM04985	NA	Multiple exons including part of 3'UTR. Covers of all isoforms (3107)
Ftz transcription factor 1 (Ftz-F1)	CG4059	3	LD34889	NA	Multiple exons and part of 3'UTR. Covers all isoforms (2270)
			LD11885	NA	First exon. Covers RB isoform (842)
Hormone receptor-like in 39 (HR39)	CG8676	4	LD45021	NA	Almost the entire transcript, Covers all isoforms (2999)
Hormone-receptor-like in 78 (HR78)	CG7199	6	GH08073	NA	The entire transcript. Covers all isoforms (2283)
Hormone receptor-like in 96 (HR96)	CG1178 3	1	GH14435	NA	The entire transcript (2834)
estrogen-related receptor (dERR)	CG7404	2	GH28308	NA	The entire transcript. Covers both isoforms (2125)
Hepatocyte nuclear factor 4 (dHNF4)	CG9310	4	RE09535	NA	The entire transcript. Covers all isoforms (3333)
seven up (svp)	CG1150 2	3	AT29920	NA	The entire transcript. Covers RA and RB isoforms (2085)
			GH08189	NA	3' UTR. Covers RC and RB isoforms (2023)
Ecdysone-induced protein 78C (E78)	CG1802 3	4	HM-E78-Ex10	up: 5'..CTTGCT GAACTTTG CCAACA down*: 5'..GCTCGC TCTTCAGT CGAGTT	Last exon, including part of 3'UTR. Covers all isoforms (764)

dissatisfaction (dsf)	CG9019	1	HM-dsf-Ex4	up: 5’..ACTCCATCCAGTCCATCTCG<br>down*: 5’..AGCAAATGCTGGTCGTTCTT	Part of exon 4 (637)
talilles (tll)	CG1378	1	HM-tll-Ex3	up: 5’..AGGGACTCTGTGTGGTGGAC<br>down*: 5’..TTGAGACCTTGTGCATCAGC	Most of exon 3 (1041)
Hormone receptor 51 (HR51)	CG16801	1	HM-DHR51-Ex6	up: 5’..ACCACCTCACCTCCTCAATG<br>down*: 5’..AAGAGCTGACGGAGGAGACA	Most of exon 6 (249)
Hormone receptor-like in 38 (HR38)	CG1864	3	HM-DHR38	up: 5’..CGACCACGTCACCTACAATG<br>down*: 5’..TAGTGCCGAATGTTGAGCAG	Last exon and part of 3‘UTR. Covers of all isoforms (644)
Hormone receptor 83 (HR83)	CG10296	1	HM-DHR83-Ex1	up: 5’..GGTGCTCCTGCTTTTTCAAG<br>down*: 5’..GCACAGGATTAGGGACTCCA	Most of exon 1 (542)
Nitric oxide synthase (Nos)	CG6713	10	HM-NOS-Ex16#	up: 5’..GAACGATTCGCTGACTGTGA<br>down*: 5’..CTTCGGAATGGCCTGCTCC	Part of exon 16. Covers 8/10 isoforms (not Nos-RJ nor Nos-RG) (1078)

*-The sequence: ‘GTAATACGACTCACTATAGGGAGACCAC’ was added before each reverse oligo sequence to add a T7 polymerase binding site.

#- For the HM-NOS-E16 probe, PCR was first performed on genomic DNA with the following oligos: up- TCGGCCTATCCAAATTTCTG and down- ACTCCTCGGTCCAAACACAC (1871 bp). The product was then amplified using the oligos indicated in the table to generate the 1078 bp template.
