# Supplementary material for: Spatial Profiling of Nuclear Receptor Transcription Patterns over the Course of Drosophila Development
Source: G3 (Bethesda). 2013 Jul 1;3(7):1177–89. doi: 10.1534/g3.113.006023 (PMC3704245; doi:10.1534/g3.113.006023)
Supplement: Supporting Information [file supp_g3.113.006023_TableS2.pdf]

Table S2 Summary of FISH expression data.

Gene	Stage	Yolk	Gut	Hindgut	Foregut	Trachea	Post. spiracles	Fat Body	Ectoderm	PNS	CNS	Muscles	Oenocytes	Amnioserosa	Salivary glands	Malpighian tubules	Lymph gland	Ring gland	Brain lobes
EcR *	E1-5	+	NA	NA	NA	NA	NA	NA	NA	NA	NA	NA	NA	NA	NA	NA	NA	NA	NA
	E6-10	+		NA	NA	NA	NA	NA				NA		+		NA	NA	NA	NA
	E11-14		+	+	NA		+			+/-	+/-			+	+	NA	NA	NA	NA
	E15-17		+	+	+	+	+		+		+/-			NA	+		NA	NA	NA
	L3	NA	+	+	+	+	NA	+	NA	NA	+	NA	NA	NA	+	+	+	+	+
Usp ~*	E1-5	+	NA	NA	NA	NA	NA	NA	NA	NA	NA	NA	NA	NA	NA	NA	NA	NA	NA
	E6-10	+	+	NA	NA	NA	NA	NA	+/-		+/-	NA		+		NA	NA	NA	NA
	E11-14	+	+		NA				+		+			+/-	+	NA	NA	NA	NA
	E15-17	+	+		+				+		+	+		NA	+	+/-	NA	NA	NA
	L3	NA	+	+	+	+	NA	+	NA	NA	+	NA	NA	NA	+	+	+	+	+
HR3	E1-5	+	NA	NA	NA	NA	NA	NA	NA	NA	NA	NA	NA	NA	NA	NA	NA	NA	NA
	E6-10	+	+	NA	NA	NA	NA	NA	+			NA		+		NA	NA	NA	NA
	E11-14	+	+	+	NA	+	+	+	+			+		+	+	NA	NA	NA	NA
	E15-17	+	+	+	+	+	+	+	+	+	+	+		NA	+		NA	NA	NA
	L3	NA	+	+	+	+	NA	+	NA	NA	+	NA	NA	NA	+	+	+	+	+
HR4	E1-5	+	NA	NA	NA	NA	NA	NA	NA	NA	NA	NA	NA	NA	NA	NA	NA	NA	NA
	E6-10			NA	NA	NA	NA	NA				NA				NA	NA	NA	NA
	E11-14	+	+	+	NA							+			+	NA	NA	NA	NA
	E15-17	+	+	+	+	+	+	+				+		NA	+		NA	NA	NA
	L3	NA				+	NA	+	NA	NA		NA	NA	NA	+	+	+	+	+
E75	E1-5	+	NA	NA	NA	NA	NA	NA	NA	NA	NA	NA	NA	NA	NA	NA	NA	NA	NA
	E6-10	+		NA	NA	NA	NA	NA				NA				NA	NA	NA	NA
	E11-14	+	+		NA	+			+	+				+	+	NA	NA	NA	NA
	E15-17	+	+		+	+	+/-			+	+		+	NA	+	+	NA	NA	NA
	L3	NA	+	+	+	+	NA	+	NA	NA	+	NA	NA	NA	+	+	+	+	+
Ftz-F1 *	E1-5	+	NA	NA	NA	NA	NA	NA	NA	NA	NA	NA	NA	NA	NA	NA	NA	NA	NA
	E6-10	+		NA	NA	NA	NA	NA				NA				NA	NA	NA	NA
	E11-14		+	+	NA	+			+	+/-	+				+	NA	NA	NA	NA
	E15-17	+	+		+	+	+		+	+/-	+			NA	+		NA	NA	NA
	L3	NA	+	+		+	NA	+	NA	NA	+	NA	NA	NA	+	+	+	+	+
HR39 *	E1-5	+	NA	NA	NA	NA	NA	NA	NA	NA	NA	NA	NA	NA	NA	NA	NA	NA	NA
	E6-10	+		NA	NA	NA	NA	NA				NA		+		NA	NA	NA	NA
	E11-14	+	+		NA			+	+							NA	NA	NA	NA
	E15-17		+		+	+	+	+	+		+			NA	+		NA	NA	NA
	L3	NA	+	+		+	NA	+	NA	NA		NA	NA	NA	+	+	+	+	
R78	E1-5	+	NA	NA	NA	NA	NA	NA	NA	NA	NA	NA	NA	NA	NA	NA	NA	NA	NA

	**E6-10**	+		NA	NA	NA	NA	NA				NA		+		NA	NA	NA	NA
	**E11-14**		+	+	NA	+			+		+				+	NA	NA	NA	NA
	**E15-17**		+	+	+	+		+/-	+		+			NA	+		NA		NA
	**L3**	NA	+	+		+	NA	+	NA	NA		NA	NA	NA	+	+	+	+	+
HR96 *	**E1-5**	+	NA	NA	NA	NA	NA	NA	NA	NA	NA	NA	NA	NA	NA	NA	NA	NA	NA
	**E6-10**	+		NA	NA	NA	NA	NA	+			NA				NA	NA	NA	NA
	**E11-14**		+	+	NA				+		+	+				NA	NA	NA	NA
	**E15-17**		+	+	+	+	+				+			NA			NA	NA	NA
	**L3**	NA	+	+		+	NA	+	NA	NA	+	NA	NA	NA	+	+	+	+	+
dERR *	**E1-5**	+	NA	NA	NA	NA	NA	NA	NA	NA	NA	NA	NA	NA	NA	NA	NA	NA	NA
	**E6-10**	+		NA	NA	NA	NA	NA				NA		+		NA	NA	NA	NA
	**E11-14**		+/-		NA						+			+	+	NA	NA	NA	NA
	**E15-17**			+	+	+	+	+/-	+		+	+		NA	+		NA	NA	NA
	**L3**	NA	+	+	+	+	NA	+	NA	NA	+	NA	NA	NA	+	+	+	+	+
dHNF4 *	**E1-5**	+	NA	NA	NA	NA	NA	NA	NA	NA	NA	NA	NA	NA	NA	NA	NA	NA	NA
	**E6-10**	+		NA	NA	NA	NA	NA	+			NA		+		NA	NA	NA	NA
	**E11-14**		+/-		NA				+				+		+	NA	NA	NA	NA
	**E15-17**		+/-		+				+		+		+	NA	+	+	NA	NA	NA
	**L3**	NA	+	+		+	NA	+	NA	NA	+	NA	NA	NA	+	+	+	+/-	+
Svp *	**E1-5**	+	NA	NA	NA	NA	NA	NA	NA	NA	NA	NA	NA	NA	NA	NA	NA	NA	NA
	**E6-10**	+		NA	NA	NA	NA	NA		+ @	+ @	NA			+	NA	NA	NA	NA
	**E11-14**		+/-		NA	+	+			+ @	+ @		+ @		+	NA	NA	NA	NA
	**E15-17**				+	+	+	+	+	+ @	+ @		+ @	NA	+ $		NA	NA	NA
	**L3**	NA	+		+	+	NA	+	NA	NA	+ @	NA	NA	NA	+	+	+	+	+
E78	**E1-5**	+ S5	NA	NA	NA	NA	NA	NA	NA	NA	NA	NA	NA	NA	NA	NA	NA	NA	NA
	**E6-10**	+	+	NA	NA	NA	NA	NA	+			NA		+		NA	NA	NA	NA
	**E11-14**	+	+		NA		+		+	+	+			+	+	NA	NA	NA	NA
	**E15-17**				+	+	+		+					NA	+	+/-	NA	NA	NA
	**L3**	NA	+	+	+	+	NA	+	NA	NA	+	NA	NA	NA	+	+	+	+	+
dsf	**E1-5**		NA	NA	NA	NA	NA	NA	NA	NA	NA	NA	NA	NA	NA	NA	NA	NA	NA
	**E6-10**			NA	NA	NA	NA	NA				NA				NA	NA	NA	NA
	**E11-14**				NA										+	NA	NA	NA	NA
	**E15-17**					+/-	+							NA	+		NA	NA	NA
	**L3**	NA	+			+/-	NA	+	NA	NA		NA	NA	NA	+	+	+	+	+
tll	**E1-5**	+/- %	NA	NA	NA	NA	NA	NA	NA	NA	NA	NA	NA	NA	NA	NA	NA	NA	NA
	**E6-10**	+/- %		NA	NA	NA	NA	NA				NA				NA	NA	NA	NA
	**E11-14**	+	+/-		NA						+				+	NA	NA	NA	NA
	**E15-17**	+	+/-		+	+			+		+			NA	+		NA	NA	NA
	**L3**	NA	+	+	+	+	NA	+	NA	NA	+	NA	NA	NA	+	+	+	+	+

HR51	**E1-5**		NA	NA	NA	NA	NA	NA	NA	NA	NA	NA	NA	NA	NA	NA	NA	NA	NA
	**E6-10**			NA	NA	NA	NA	NA				NA				NA	NA	NA	NA
	**E11-14**				NA	+	+ (^)		+ (#)	+						NA	NA	NA	NA
	**E15-17**				+	+	+ (^)		+ (#)					NA	+		NA	NA	NA
	**L3**	NA				+/-	NA	+	NA	NA		NA	NA	NA	+	+	+	+	+
HR38	**E1-5**		NA	NA	NA	NA	NA	NA	NA	NA	NA	NA	NA	NA	NA	NA	NA	NA	NA
	**E6-10**			NA	NA	NA	NA	NA				NA				NA	NA	NA	NA
	**E11-14**				NA											NA	NA	NA	NA
	**E15-17**					+	+		+/-					NA	+		NA	NA	NA
	**L3**	NA				+	NA	+	NA	NA	+	NA	NA	NA			+	+	
HR83	**E1-5**		NA	NA	NA	NA	NA	NA	NA	NA	NA	NA	NA	NA	NA	NA	NA	NA	NA
	**E6-10**			NA	NA	NA	NA	NA				NA				NA	NA	NA	NA
	**E11-14**				NA										+	NA	NA	NA	NA
	**E15-17**					+								NA	+		NA	NA	NA
	**L3**	NA				+/-	NA	+	NA	NA		NA	NA	NA	+	+	+	+	
Nos	**E1-5**	+/-	NA	NA	NA	NA	NA	NA	NA	NA	NA	NA	NA	NA	NA	NA	NA	NA	NA
	**E6-10**	+	+	NA	NA	NA	NA	NA				NA				NA	NA	NA	NA
	**E11-14**	+	+	+	NA											NA	NA	NA	NA
	**E15-17**	+	+	+	+	+	+	+			+	+		NA	+		NA	NA	NA
	**L3**	NA	+		+	+	NA	+	NA	NA		NA	NA	NA	+	+	+	+	+

+: expressed, NA: not applicable, *: "fluffy" basal localization, ^: posterior spiracle outer layer, #: stripes, s5: stage 5 only, %: *tll* is zygotically expressed at this stage, some transcript ends up at the yolk. ~: low ubiquitous expression, @: specific to *svp*-AT29920 probe, $: specific to *svp*-GH0819 probe. Shaded grey: matches spatial activities noted in Palanker *et al* 2006.
