# Supplementary material for: Spatial Profiling of Nuclear Receptor Transcription Patterns over the Course of Drosophila Development
Source: G3 (Bethesda). 2013 Jul 1;3(7):1177–89. doi: 10.1534/g3.113.006023 (PMC3704245; doi:10.1534/g3.113.006023)
Supplement: Supporting Information [file supp_g3.113.006023_FigureS1.pdf]

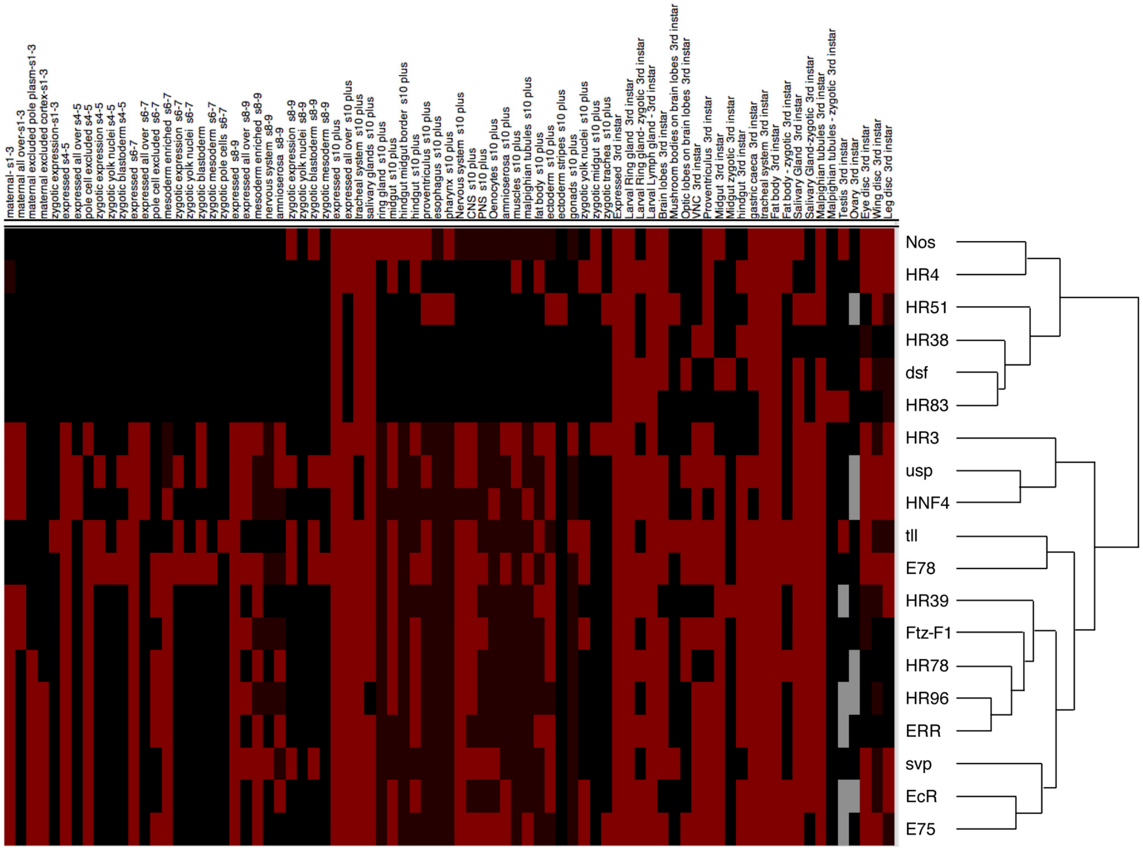

Figure S1 Expression pattern relationships. Cluster comparisons between all 18 nuclear receptors and NOS tissue expression patterns was performed using 'Gene Cluster' and 'Java TreeView' software. Gene identities are listed to the left of each row, and terms for the tissues and stages analyzed above each column. Bright red = strong expression; dark red = lower expression; black = no expression; grey = no data. The tree on the right shows grouping by overall similarity.
