# Supplementary material for: Spatial Profiling of Nuclear Receptor Transcription Patterns over the Course of Drosophila Development
Source: G3 (Bethesda). 2013 Jul 1;3(7):1177–89. doi: 10.1534/g3.113.006023 (PMC3704245; doi:10.1534/g3.113.006023)
Supplement: Supporting Information [file supp_g3.113.006023_TableS3.pdf]

Table S3 Summary of FISH subcellular patterns. A summary of all the subcellular RNA patterns found in this study. The developmental stages are shown at the left of the Table. +: pattern present, -: pattern absent.

Stage	Term	*EcR*	*usp*	*HR3*	*HR4*	*E75*	*Ftz-F1*	*HR39*	*HR78*	*HR96*	*dERR*	*dHNF4*	*svp*	*E78*	*dsf*	*tll*	*HR51*	*HR38*	*HR83*	*Nos*
Embryo 1-3	Pole plasm excluded	+	-	-	-	+	-	-	+	+	+	-	+	-	-	-	-	-	-	-
	Cortex excluded	+	-	-	-	+	-	-	-	+	+	-	+	-	-	-	-	-	-	-
	Small foci	-	-	+	-	+	-	-	-	-	-	+	-	-	-	-	-	-	-	-
	Enriched around yolk nuclei	+	-	-	-	+	-	-	+	-	+	+	-	-	-	-	-	-	-	-
Embryo 4-5	Pole cell excluded	+	-	+	-	+	+	+	+	+	+	-	+	+	-	+	-	-	-	-
	Apical excluded	+	-	+	-	+	+	+	+	+	+	-	+	+	-	-	-	-	-	-
	Basal excluded	+	-	+	-	+	+	+	+	+	+	-	+	+	-	-	-	-	-	-
	Cortex excluded	+	-	+	-	+	+	+	+	+	+	-	+	+	-	-	-	-	-	-
	Apical enriched	-	-	-	-	-	-	-	-	-	-	-	-	-	-	+	-	-	-	-
	Basal enriched	-	-	-	-	-	-	-	-	-	-	-	-	-	-	+	-	-	-	-
	Enriched around yolk nuclei	+	-	-	-	+	+	-	+	+	+	-	+	+	-	-	-	-	-	+
	Perinuclear- yolk nuclei	-	-	-	-	-	-	-	+	-	+	-	-	-	-	-	-	-	-	-
	Small foci	-	+	-	-	+	-	-	-	-	-	+	-	+	-	-	-	-	-	-
Embryo 6-7	Pole cell excluded	-	-	-	-	+	-	+	+	+	+	-	-	+	-	-	-	-	-	-
	Apical excluded	+	-	-	-	+	-	+	+	+	+	-	-	-	-	-	-	-	-	-
	Basal excluded	-	-	-	-	-	-	-	-	-	-	-	-	-	-	-	-	-	-	-
	Apical enriched	-	-	-	-	-	-	-	-	-	-	-	-	-	-	+	-	-	-	-
	Basal enriched	+	-	+	-	+	-	+	+	+	+	+	+	-	-	+	-	-	-	-
	Enriched around yolk nuclei	+	-	-	-	+	-	+	+	+	+	+	+	-	-	-	-	-	-	+
	Perinuclear- yolk nuclei	-	-	-	-	-	-	-	+	+	-	-	-	-	-	-	-	-	-	-
	Perinuclear- pole cells	-	-	-	-	-	-	-	-	-	-	-	-	-	-	+	-	-	-	-
	Small foci	+	-	+	-	+	+	+	-	+	+	+	+	+	-	+	-	-	-	-
Embryo 8-9	Apical enriched	-	-	-	-	+	-	-	-	-	-	-	-	-	-	-	-	-	-	-
	Basal enriched	+	+	-	-	-	-	+	+	+	+	-	-	-	-	+	-	-	-	+
	Fluffy basal	+	-	-	-	-	-	-	+	-	-	-	-	-	-	-	-	-	-	-
	Enriched around yolk nuclei	-	-	-	-	+	-	-	+	-	-	-	-	-	-	-	-	-	-	+
	Perinuclear- yolk nuclei	-	-	-	-	-	-	-	-	-	-	-	-	-	-	-	-	-	-	+/-
	Perinuclear - blastoderm nuclei	+	-	-	-	-	-	-	-	-	-	-	-	-	-	+	-	-	-	+
	Perinuclear- pole cells	-	-	-	-	-	-	-	-	-	-	-	-	-	-	+	-	-	-	-
	Small foci	+	+	+	-	+	-	+	-	-	-	+	+	+	-	-	-	-	-	-
	Amnioserosa - foci	+	-	+	-	-	-	-	+	-	-	-	-	-	-	-	-	-	-	-
Embryo 10-17	Tracheal lumen*	+	+	+	+	-	+	+	+	+	+	-	+	+	+	+	+	+	+	+
	Tracheal system - specific area	-	-	+	-	+	-	-	-	-	-	-	-	-	-	-	+	-	-	-
	Salivary gland - basal	+	+	-	-	+	-	-	+	-	-	-	+	-	-	-	-	-	-	-
	Salivary gland - apical (lumen)*	+	+	+	+	+	+	-	+	-	+	+	+	+	-	+	-	+	-	+
	Hindgut - apical	-	-	-	-	-	-	-	-	-	+	-	-	-	-	-	-	-	-	+

	Hindgut - basal	-	-	-	-	-	-	-	+	-	-	-	-	-	-	-	-	-	-	-
	Enriched around yolk nuclei	-	-	-	-	+	-	-	-	-	-	-	-	+	-	+	-	-	-	-
	Fluffy basal	+	+	-	-	+	+	+	+	+	+	-	+	-	-	-	-	-	-	-
	Small foci	+	-	+	-	+	-	-	-	-	+	+	-	+	-	-	-	-	-	-
Larva 3rd instar	Ring gland - foci	+	+	+	-	+	+	-	+	+	+	-	+	+	+	+	+	+	+	+
	Ring gland - perinuclear	-	-	-	-	+	-	-	-	-	-	-	-	-	+	-	-	-	-	-
	Ring gland - perinuclear diffuse	-	+	-	-	-	-	-	+	+	-	-	-	-	-	+	-	-	-	-
	Brain lobe - foci	-	+	-	-	+	+	-	-	-	+	+	+	+	+	-	-	+	-	+
	Midgut - foci	+	+	-	-	+	-	+	-	-	-	+	+	-	+	+	-	-	-	+
	Midgut - perinuclear half moon	-	-	-	-	+	-	-	-	-	-	-	-	-	-	-	-	-	-	-
	Hindgut - foci	+	-	-	-	+	-	-	-	+	-	-	-	-	-	+	-	-	-	-
	Gastric caeca - foci	+	-	-	+	-	-	+	-	-	-	-	+	-	+	-	+	-	-	+
	Fat body - foci	+	+	+	+	-	+	+	+	+	+	+	+	+	+	+	+	+	+	+
	Fat body - perinuclear half moon	-	-	-	-	+	-	-	-	-	-	-	-	-	-	-	-	-	-	-
	Salivary gland - foci	-	+	+	+	-	+	-	-	+	-	+	+	+	-	+	+	-	-	+
	Salivary gland - perinuclear half moon tight	-	-	-	-	+	+	+	+	+	-	-	+	-	-	+	-	-	-	-
	Salivary gland - perinuclear half moon loose	+	-	-	-	-	-	+	+	-	+	+	+	+	-	+	-	-	-	-
	Salivary gland - perinuclear full	-	-	-	-	-	-	-	-	-	-	-	-	+	-	-	-	-	-	-
	Malpighian tubule - foci	+	+	-	+	+	+	+	+	+	-	+	+	+	+	+	+	-	+	+
	Malpighian tubule - perinuclear half moon	-	-	-	-	+	-	-	+	-	-	-	-	-	-	-	-	-	-	-
